# Hydrological processing of salinity and nitrate in the Salinas Valley agricultural watershed

**DOI:** 10.1007/s10661-020-08811-3

**Published:** 2021-05-14

**Authors:** Prudentia Zikalala, Isaya Kisekka, Mark Grismer

**Affiliations:** 1 Department of Land, Air, and Water Resources, University of California, One Shield Avenue, Davis, CA USA; 2 Department of Land, Air, and Water Resources & Biological and Agricultural Engineering, University of California, Davis, One Shield Avenue, Davis, CA USA

**Keywords:** Salinity, Nitrate, Agricultural pollution, Water quality, Statistics, Time-series analysis

## Abstract

Regime shifts of major salinity constituents (Ca, Mg, Na, K, SO_4_, Cl, HCO_3_, and NO_3_) in the lower Salinas River, an agricultural ecosystem, can have major impacts on ecosystem services central to continued agricultural production in the region. Regime shifts are large, persistent, and often abrupt changes in the structure and dynamics of social-ecological systems that occur when there is a reorganization of the dominant feedbacks in the system. Monitoring information on changes in the system state, controlling variables, and feedbacks is a crucial contributor to applying sustainability and ecosystem resilience at an operational level. To better understand the factors driving salinization of the lower Salinas River on the central coast of California, we examined a 27-year record of concentrations of major salinity constituents in the river. Although limited in providing an understanding of solute flux behavior during storm events, long-term “grab sampling” datasets with accompanying stream discharges can be used to estimate the actual history of concentrations and fluxes. We developed new concentration–discharge relationships to evaluate the dynamics of chemical weathering, hydrological processes, and agricultural practices in the watershed. Examinations of long-term records of surface water and groundwater salinity are required to provide both understanding and perspective towards managing salinity in arid and semi-arid regions while also enabling determination of the influence of external climatic variability and internal drivers in the system. We found that rock weathering is the main source of Ca, Mg, Na, HCO_3_, and SO_4_ in the river that further enables ion exchange between Ca, Mg, and Na. River concentrations of K, NO_3_, and Cl were associated with human activities while agricultural practices were the major source of K and NO_3_. A more direct anthropogenic positive trend in NO_3_ that has persisted since the mid-1990s is associated with the lag or memory effects of field cropping and use of flood irrigation. Event to inter-year scale patterns in the lower Salinas River salinity are further controlled by antecedent hydrologic conditions. This study underscores the importance of obtaining long-term monitoring records towards understanding watershed changes-of-state and time constants on the range of driving processes.

## Introduction

Information on salinity processes operating within a watershed is critical towards evaluating ecosystem services or ES (including agricultural production, freshwater used for irrigation, municipal water supplies, pollution dilution, and recreation) of the river system. Modification of the water cycle through agriculture can lead to changes in the magnitude and timing of water flows, contributing to shifts in river pollution states (regime shift) in downstream water bodies, soil moisture regimes, and microclimates (Gordon et al. 2008). These changes can have enormous consequences for long-term food production. In most parts of the world, the regime shifts to which a particular region may be vulnerable under different land uses or under conditions such as a changing climate are largely unknown, as are the effects of potential regime shifts on ecosystems, ecosystem services, and human well-being. Such information is critical to sustainable development planning and to assessments of social and ecological resilience, which are increasingly central to development policy (Reyers et al. 2018).

Several studies underscore the importance of studying and interpreting patterns of human modification in the landscape to understand deeply the consequences of human intervention in the past and to better plan engineered responses to future challenges (Reinette Biggs et al. 2012). Wagener et al. (2010) and Grismer (2019) have called for a new paradigm for hydrologic science that includes human-induced changes as integral to the overall hydrologic system. Recently, Pande and Sivapalan (2017), M. Sivapalan et al. (2014), Kandasamy et al. (2014), and Murugesu Sivapalan et al. (2012) proposed the sub-field ‘socio-hydrology’ with “a focus on the understanding, interpretation and prediction of the flows and stocks in the human-modified water cycle at multiple scales, with explicit inclusion of the two-way feedbacks between human and water systems” to address these challenges.

Changes in ecosystems and social-ecological systems (SESs) are usually experienced as relatively slow and incremental, but from time-to-time dramatically large, persistent, and often unexpected changes take place. Such large, persistent changes are commonly referred to as regime shifts (Scheffer et al. 2001; Reinette Biggs et al. 2012). Understanding of how agricultural modifications of the hydrological cycle regulate the prevalence and severity of surprising nonlinear change is lacking (*Comprehensive Assessment of Water Management in Agriculture.* 2007; Carpenter et al. 2006). However, a growing body of evidence suggests that agricultural modification of the quantity and quality of hydrological flows can increase the risk of ecological regime shifts (Walker and Meyers 2004; Biggs et al. 2018). Applying ecosystem resilience and sustainability at an operational level requires understanding the linkages between socioeconomic and natural systems (Mavrommati et al. 2014; Reinette Biggs et al. 2012). Using literature, Reinette Biggs et al. (2012) identified seven principles for enhancing the resilience of desired ES in the face of disturbance and ongoing change in SES. Their principle 3 outlined a need to manage slow variables and feedbacks for maintaining SES regimes that underlie the production of desired ES. Applying this principle, however, requires understanding of the possible regime shifts and their consequences as well as the key factors that trigger such regime shifts. Here, we examined long-term records of surface water salinity to produce both understanding and perspective toward managing salinity in arid and semi-arid regions while also enabling the study of the influences of external climatic variability and internal drivers in the system.

Agriculture is now the largest anthropogenic disturbance in the watershed in terms of area, followed by urbanization and dam emplacement (J. G. Thompson and Reynolds 2002). As well, J. G. Thompson and Reynolds (2002) noted the major project goals of the development of the Salinas Valley Basin Management Plan by the Monterey County Water Resources Agency since 1992 was to sustain and even further develop profitable agricultural enterprises. As such, our aim was to quantify the dynamic relationships between solute concentration and stream discharge to improve our understanding of solute transport pathways and active source areas in the Salinas watershed. Elucidation of temporal dependence in solute dynamics of a highly developed, semi-arid basin is a forensic exercise of implicating and eliminating a host of potential contributing factors, or controls. When discrete controls on solute dynamics are discovered in a developed watershed, it is often the result of scenarios where proportionally large areal disturbances have dominated the solute responses; in this way, urbanization (Beaulieu et al. 2012) and agriculture (Kaushal et al. 2005) have been found to exert significant control on stream solute fluxes.

The 1972 Clean Water Act (as amended in 1987) is the principal instrument of non-point pollution reform in the USA. This Act mandates ‘listing’ (i.e., 303d list) of all impaired water bodies in the USA, and development of Total Maximum Daily Load (TMDL) plans to address the impairment. The lower Salinas River is 303d listed for salinity (total dissolved solids and chloride) and nutrients (ammonia and nitrate). Furthermore, the 2015 Senate Bill 390 and later California Superior Court ruling required the Central Coast Water Quality Control Board (Regional Board) to develop more comprehensive water quality protections. In 2017, the Regional Board adopted conditional waivers for dischargers from irrigated lands which required monitoring, reporting, completion of annual compliance forms, development of irrigation, nutrient management, and water quality buffer plans depending on one of three applicable ‘Tiers’ of land use. These are stringent requirements that are costly for farmers and to be successful require that farmers understand and trust that their efforts will result in improved water quality overtime (Drevno 2018). Studies that provide quantitative links between land management practices and water quality outcomes are therefore essential.

Solute fluxes in streams from developed watersheds in semi-arid environments are influenced by natural and human-induced changes to the land surface that interact with variable climatic regimes. The causes of saline influxes to rivers can be due to natural processes or to secondary processes arising from anthropogenic changes in the catchment. Secondary salinization arises from a number of factors such as changing land use and rising saline water tables discharging into the rivers and saline soil pools. For example, saline flows in the Colorado River have increased over the last 100 years (Western et al. 1996; Peck and Hatton 2003; Lamontagne et al. 2005; Jolly et al. 2008; Shirinian-Orlando and Uchrin 2000) presumably associated with water diversions and upstream developments. Natural salinization processes in semi-arid zone catchments are generally considered to result from dry and wet deposition of marine salts by rainfall (Simpson and Herczeg 1994; Allison et al. 1985), re-suspended regional dust (Simpson and Herczeg 1994), and rock weathering (Horton et al. 1999; Moreira-Nordemann 1984). The latter concentrates in the root and unsaturated zones eventually seeping to deeper groundwater or is collected by artificial subsurface drainage systems. The complexity of the processes affecting surface to soil-water systems makes it difficult to unravel the extent of natural salinization from the accelerated processes caused by anthropogenic influences.

Long-term monitoring indicates that solute concentration behavior often exhibits temporal dependence over event to inter-decadal time scales particularly in arid to semi-arid climates (Murphy et al. 2014; Worrall et al. 2003; Burt and Worrall 2009). Factors affecting watershed-scale solute production operate over a wide range of time scales, with even seemingly discrete events generating legacy effects that may endure for years. That is, salinity and nitrate concentrations in the catchment may be responding to land use changes from decades prior. In this context, salinity and nitrate management measures introduced historically cannot be judged successful without an ability to communicate water quality changes, particularly changes in forcing factors (internal or external) over time that then affect watershed scale solute production and transport.

In a study of streamflow nitrate concentrations within a small agricultural catchment of southwest England, Burt and Worrall (2009) observed changes in the system state over time and concluded that there was non-stationarity in long-time series. They suggested that long-term records are required to understand the time constants of a range of driving processes. Event to inter-annual scale concentration behavior in river systems is known to be influenced by antecedent hydrologic conditions, whereby previous hydrologic activity regulates the concentration-discharge relationship (Murphy et al. 2014). Responses of solute concentrations to fluctuations in streamflow have been observed to vary between sites, to vary for specific species, and to vary for specific storms. Magnitudes of antecedent storm events, mixing of event water with groundwater, long periods of low flow, variable chemistry of “old” water, and catchment flushing times have all been related to perturbations of long-term hydro-geochemical response of a catchment (Butturini et al. 2006; Edwards 1973; Hill 1993; Kirchner 2003; Rademacher et al. 2005; Walling and Foster 1975; Creed and Band 1998). Biron et al. (1999) studied the effect of antecedent soil-moisture conditions on stream water quality and found that with dry antecedent conditions, there was a general decrease in solute concentrations with time, whereas concentrations remained about the same under wet conditions. Butturini and Sabater (2000) and Piñol et al. (1992) studied effects of rainfall variability and consequent flushing of soils on stream chemistry and found that changes in stream fluxes are to the first order, dependent upon changes in hydrology.

Temporal variability in stream salinity in the developed Salinas Valley watersheds was determined from “grab sampling” datasets of major salinity components (Ca, Mg, Na, K, SO_4_, Cl, HCO_3_, and NO_3_) and associated discharges and a complete record (hourly) of daily stream discharge from 1977 to 2013. Although limited in providing an understanding of solute flux behavior during storm events (House and Warwick 1998; Vaughan et al. 2017), long-term “grab sampling” datasets with accompanying instantaneous discharge and mean daily stream discharge datasets for the complete record can be used to estimate the actual history of concentrations and fluxes using concentration–discharge relationships. Continuous monitoring of stream water quality is expensive; however, frequent flow monitoring coupled with long-term stream water quality “grab sampling” is widely available especially in the USGS database. We sought to apply methods that can use these available datasets as diagnostic tools regarding the changes in stream solute concentrations taking place in the watershed of interest. To improve water quality, there is great value in developing and using data analysis methods aimed at deriving the greatest possible amount of information from the data that are collected, particularly related to changes in water quality over time.

The overarching objective of this paper was to provide understanding of the stream solute-concentration dynamics in the lower Salinas River over inter-decadal time scales. Our aim was to disentangle multiple controls on river salinity at a regional scale, and to infer mechanisms behind fluctuations of solute concentrations in the lower Salinas River over time. The goals of this study were six-fold to:

1. analyze concentration–discharge (C–Q) relationships based on three models (a) linear regressions, (b) locally weighted scatter-plot smoother (LOESS) regression techniques, and (c) weighted regressions on time, discharge, and season (WRTDS model; Hirsch et al. (2010)).
2. identify and describe the inter-annual patterns of the major salinity constituents’ concentration–discharge behavior. That is, to identify regime shifts in solute concentrations in the river (“Regime shifts in solute concentrations: inter-annual variability”).
3. identify hydrogeochemical processes responsible for the production of different solute concentrations over time in the lower Salinas River (“Hydrogeochemical processes controlling solute concentrations over time”).
4. identify flow regimes that have controls on shifts in solute concentrations (“Assessment of the effects of stream flow on shifts in river solute concentrations”).
5. examine the effects of antecedent flow conditions on solute concentrations in the river (“Effect of antecedent hydrologic conditions on C–Q residuals”).
6. describe the factors controlling relationships between river nitrate concentrations and antecedent flow climate conditions, basin characteristics, and management practices and how these relationships evolve in time (“Relationship between NO3-Q residuals, climate and land use variables”).

Recent studies highlight the significance of nitrate contamination of drinking water sources (Harter et al. 2012); as such, we dedicated a section to the understanding dynamics of NO_3_ concentrations in the river. Through these analyses, we developed a more holistic perspective of how basin characteristics including water yield, evaporative demands, base flow, land use, and land management practices affected the temporal evolution of Salinas River salinity during the past two and a half decades.

## Materials and methods

### Study area

**Fig. 1:**
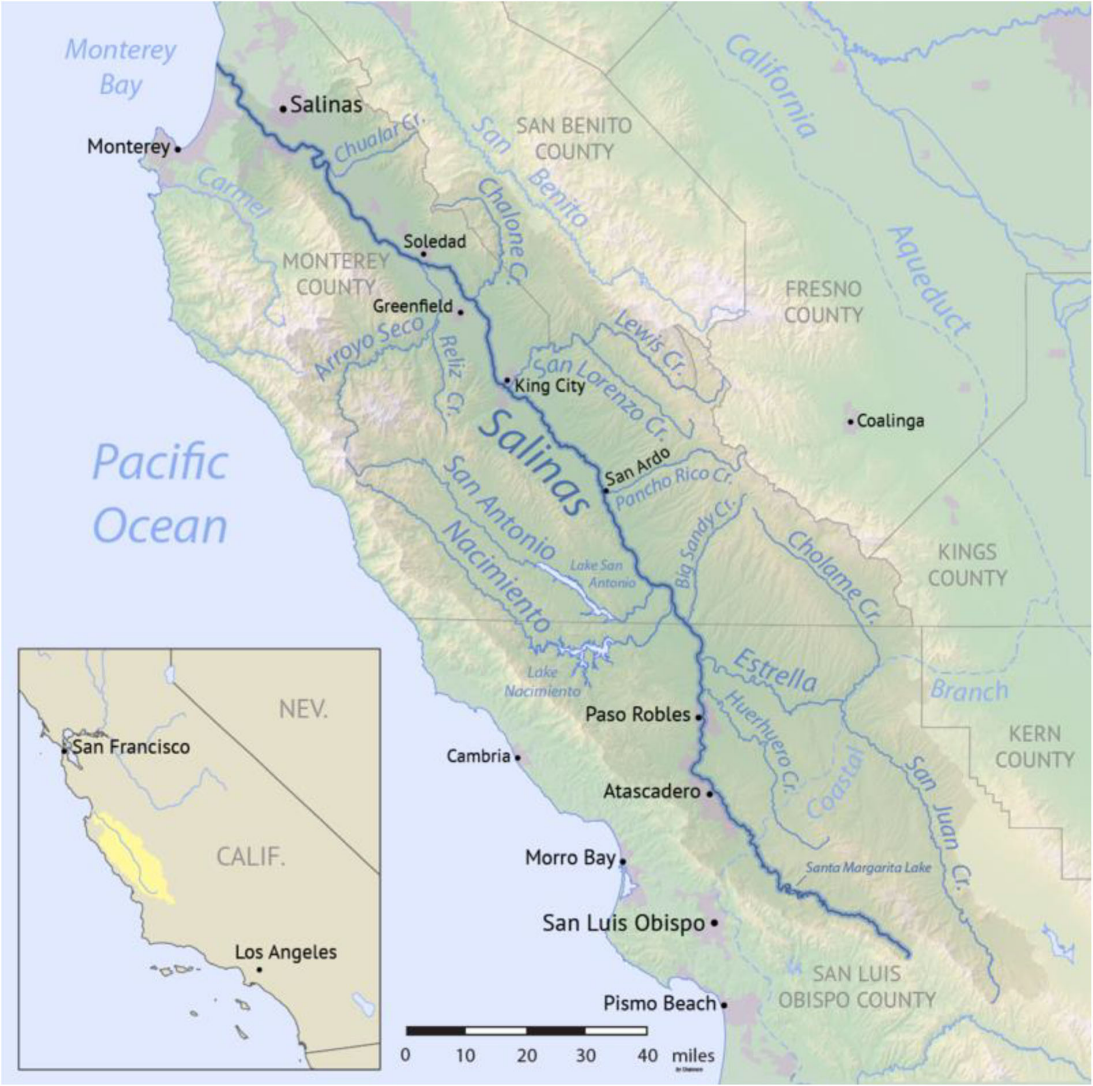
Salinas Valley watershed. Source: https://creativecommons.org/licenses/by-sa/4.0/

Located in the Coast Ranges of central California, the Salinas Valley area is a geomorphic province between the Great Valley and the Pacific Ocean (Fig. 1). The Salinas River watershed covers 11,422 km^2^ in Monterey and San Luis Obispo counties and a small portion in San Benito County. The climate is Mediterranean semi-arid with warm, dry summers and cool, moist winters. The long-term (1895 to 2015) average temperature in Monterey County was 14.7 °C (±3.9%). Average annual precipitation was 470 mm (±35.6%). Precipitation generally increases with altitude and decreases from north to south (Kulongoski and Belitz 2011).

Mountainous highlands of the watershed are mostly composed of Mesozoic-aged sedimentary and metasedimentary rock with some igneous intrusions, while the northern extent of the main stem valley floor is Tertiary and younger alluvial fill (Nutter 1901; Durham 1974). Maximum relief in the basin is ~1900 m and average watershed bounding ridge heights are 750 m to the NE and 1200 m in the SW, with ridge crest height generally decreasing toward the mouth of the Salinas. Land cover in the Salinas watershed largely follows local relief, with steep forested terrain giving way downslope to chaparral/scrub in the wetter western hills and grassland in the drier eastern hills (Farnsworth and Milliman 2003). The Salinas River originates in the south near Santa Margarita in San Luis Obispo County and flows northwest from an elevation of ~274 to ~61 m near Greenfield along US Highway 101 approximately 32 km to the Pacific Ocean at Monterey Bay, discharging north of the City of Marina. The Salinas, from its junction with the Estrella River to the junction with the San Antonio River, flows on a narrow flood plain (~0.4 km wide). Nearby hills to the east that are 213 to 305 m above the flood plain have blunt steep faces and numerous slides; both features suggest erosion at the base of the hills by the Salinas River. North of its junction with the San Antonio River, the Salinas flows through a narrow canyon. Opposite the mouth of San Lorenzo Creek, the Salinas River is on the west side of the valley; farther north opposite the mouth of the Arroyo Seco, it is on the east.

Mean annual discharge of the Salinas River ranges from 350 million m^3^/year near Chualar and near ~432 million m^3^/year near Bradley below Nacimiento dam. Streamflow near Bradley represents environmental and groundwater recharge releases from the Nacimiento and San Antonio reservoirs with most flow occurring during November through March. These major reservoirs, located along the Nacimiento River and the San Antonio River, were completed in 1957 and 1967, respectively. The Salinas is a losing stream with naturally transient flow and no surface water passing through the lower reaches for much of the summer. The aquifers in the alluvial valley are overdrafted for agricultural production, causing saltwater intrusion. During the spring and summer, the Nacimiento and San Antonio reservoirs are operated by Monterey County Water Resources Agency (MCWRA) to maintain required fish bypass flows at the Salinas River Diversion Facility, while maximizing recharge to the groundwater basin via the Salinas River bed (MCWRA 2008).

Today, the hydrological conditions of the Salinas River differ from that historically due to changes in basin land use and flow regulation. By 1901, groundwater pumping was underway, with wells drawing water from as deep as 75 m below the ground surface and lowering the water table below the ground by 3–5 m (Lapham and Heileman 1901). Valley bottoms were mostly converted to irrigated agriculture during the past century with a small proportion of urbanization. The Salinas Valley is primarily cropland, known as “America’s Salad Bowl” due to the prominence of vegetables and greens grown in the region while numerous vineyards are also present. Land use or cover in the watershed varies greatly by location. The largest urban area in the Salinas River watershed is the City of Salinas, located in the lower portion of the watershed. The City population in 2010 was 150,441 while the six other smaller cities in the area (Atascadero, Soledad, Greenfield, King City, Paso Robles) have populations under 30,000 (Census 2010). Anderson (2000) details the parallel histories in the Salinas Valley of land value and ownership, irrigation practices and areas, soil management, transportation, processing technology, and trade. Introduction of the turbine pump with a capacity to lift hundreds of feet in 1924 revolutionized groundwater extraction in the Valley. In 1889, only some 170 ha of the Valley was irrigated using historic ditch systems, less than 0.2% of the total cropped area. Crops included barley, wheat, beans, sugar beets and potatoes, and there were about 56,722 grazing cattle. By 1939, 30% of 139,400-ha agricultural production employed irrigation; about 56,600 ha consisted of vegetable crops introduced A total of ~1900 and 118,000 cattle grazed the Valley. Just 4 decades later, by 1980 dams were built on the Nacimiento and San Antonio rivers to supplement water supply and dam releases were used to recharge groundwater. By 1980, about half of the 181,300 ha in agricultural production was under irrigation, with grape production dramatically increasing from 60 ha in 1939 to 12,166 ha in 1980. Later, growers adopted water conservation technologies such as drip, sprinkler, and surge-flood irrigation presumably to reduce the farm water ‘footprint’ and water quality impacts associated with runoff from irrigation.

### Data

**Fig. 2:**
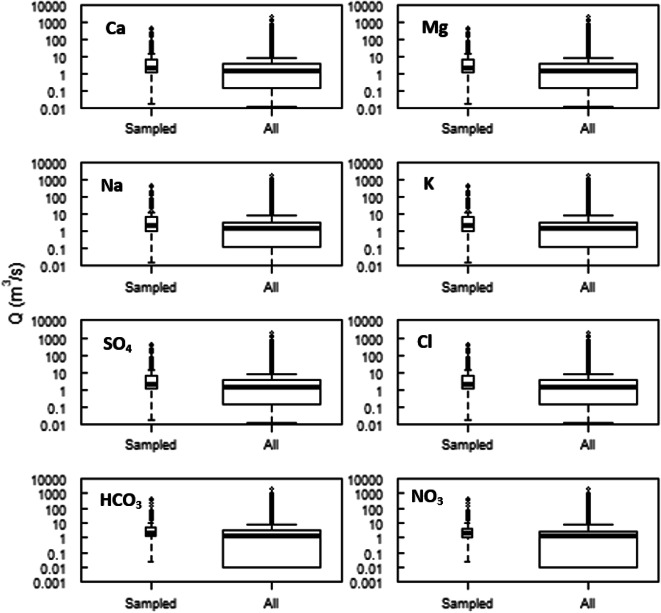
Box plot of discharge at which samples were collected and daily mean discharge values for the entire period of record at Chualar 1977–2013

We used historical data from the US Geological Survey (USGS) sampling of the lower Salinas River at the Chualar station that captures drainage from an area of ~10,469 km^2^ (USGS station #11152300). Concentrations of major salinity constituents Ca, Mg, Na, K, SO_4_, and Cl were available from 1977 to 2013; concentrations for HCO_3_ were available from 1989 to 2013 and for NO_3_ from 1986 to 2013. On average, there were five sampling events per year. All USGS-measured (grab sample) solute concentrations were associated with instantaneous discharge values. These were combined with the daily-hourly discharge values for the site over the entire period. To ensure that disparities in discharge and concentration sampling frequencies do not bias the data record, grab sampling data is needed across the full flow duration hydrographs. We evaluated the flow and grab sampling information and Fig. 2 shows discharge-concentration plots for the different solutes. Boxplots of discharge at which samples in the data were collected and that of daily mean discharge for the entire period are similar in terms of median, upper and lower quartiles for Ca, Mg, SO_4_, HCO_3_, and NO_3_. However, there is under-sampling at low flows for Na and K. Furthermore, we verified the general anion-cation balance for all samples to ensure sampling data chemical consistency.

Monthly precipitation, potential, and actual evapotranspiration records were estimated from the USGS California-Basin Characterization Model (BCM). The BCM applies a monthly regional water balance model to simulate hydrologic response to climate at the grid spatial resolution of 270m (Flint et al. 2013). Monthly estimates of climatic water deficit (CWD), potential evapotranspiration (PET), and actual evapotranspiration (AET) were extracted from this model. CWD is the evaporative demand that exceeds available water and is calculated as PET minus AET. In this model, available water occupies the soil profile where it will become AET and may also result in runoff or recharge depending on the soil storage and permeability of the underlying bedrock. CWD is strongly correlated with the distribution of vegetation in a landscape. CWD accumulates the deficit across the season and thus is an indicator of the irrigation required to make up the seasonal water deficit (Stephenson 1998).

**Fig. 3:**
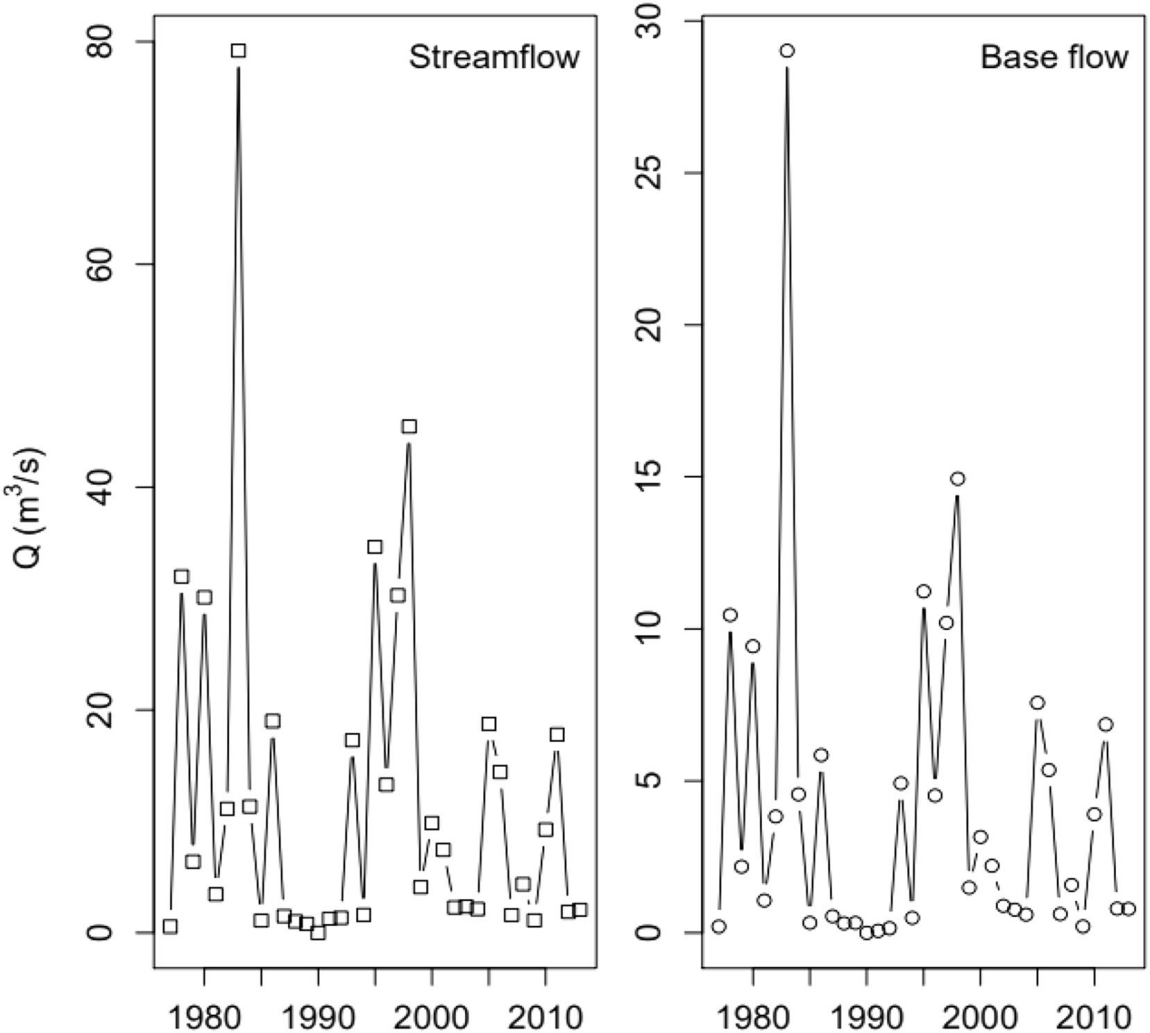
Annual average baseflow from streamflow based on the mean daily streamflow

Base flows were estimated from average daily streamflow data using WHAT, the Web-based Hydrograph Analysis Tool (Lim et al. 2005). WHAT is implemented with the Recursive Digital Filter Method and the maximum Base Flow Index calibrated to be equivalent to the base flow indices reported by the USGS survey for Salinas River at Chualar (0.338) (Wolock 2003). Trends in streamflow and simulated base flow are shown in Fig. 3.

Spatial coverage of crops by year from 1929 to 2013 was obtained from the Monterey County Agricultural Commissioner’s Office Crop Reports (Monterey County 2016; MCAC 1929-2016). Data on the areal coverage agriculture by irrigation and conservation techniques from 1993 to 2013 were extracted from groundwater extraction summary reports from the Monterey County Water Resources Agency (MCWRA 2015). The MCWRA graciously provided data on water deliveries from the Recycled Water Project, Salinas River Diversion Facility, and groundwater extractions for irrigation uses from 1949 to 2016.

### Computation and data analysis

**Fig. 4:**
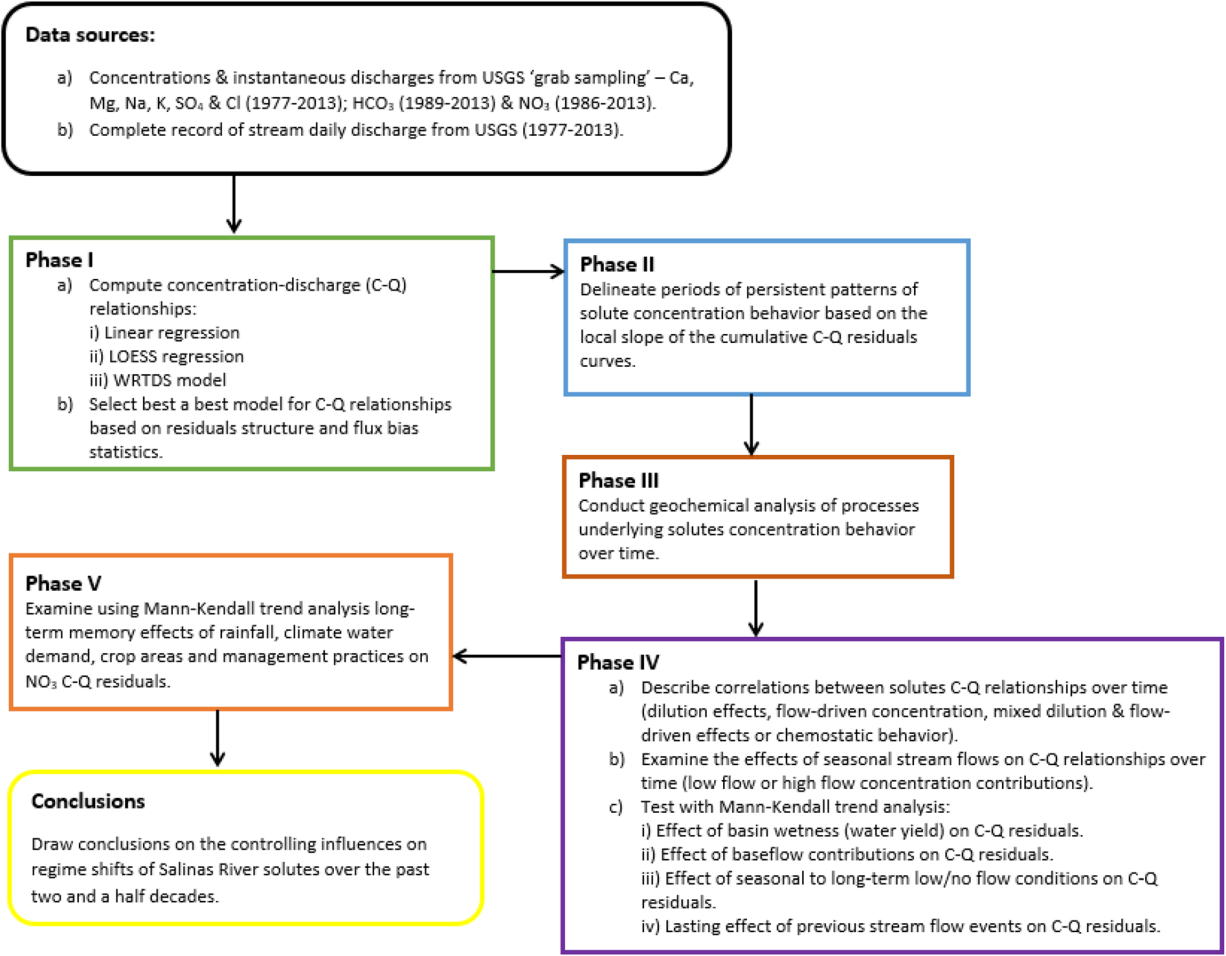
Analysis approach to determine controlling influences on solute concentrations over time

We combined several analysis approaches to gain insights into long-term controls on the concentrations of major salinity constituents in the lower Salinas River as graphically outlined in Fig. 4. The first phase of this study involved using the available solute concentrations (C) and associated flow (Q) data to model dependence of different solute C on Q using the linear regression, LOESS regression techniques, and Weighted Regressions on Time, Discharge, and Season (WRTDS) models. Solute rating curves were computed for the entire sampling period—1977 to 2013 for concentrations of Ca, Mg, Na, K, SO_4_, and Cl: 1988 to 2013 for HCO_3_ concentrations and 1986 to 2013 for NO_3_ concentrations. Based on the residual structure and flux bias statistics, a best model was selected (here, the WRTDS).

Residuals between actual measured values and those determined from the best C–Q relationships were then used to describe changes in these relationships over time. We calculated residuals by subtracting the expected (from each rating curve) from observed sample values and then accumulated these over times. These residuals reveal systematic departures in sample C behavior from that of the rating curve model that is assumed to capture longer-term average behavior. Residuals from the model represent a combination of measurement error, inadequacies of the model’s functional form, estimation error of the coefficients, and the influence of other variables that are not considered by the model. Thus, positive residuals indicate higher-than-anticipated observed concentrations, while negative residuals indicate a lower-than anticipated observed concentration. The second phase involved the delineation of periods of persistent patterns in solute concentration behavior (regime shifts) by sequentially summing, that is, accumulating C–Q WRTDS residuals over time. Periods of persistent positive or negative behavior were identified based on the local slope of the cumulative residual curve, with persistent positive or negative values identified by positive or negative slopes maintained over time.

A third phase involved analysis of hydro-geochemical processing underlying solute behavior within the identified persistent periods of high or low. This was accomplished using compositional relations and ionic ratios among major dissolved species to gain insights into possible origins of water quality.

Task 4 has two parts; the first part considers the load dynamics from discharge variability over the different periods to describe correlations between solute concentrations and river discharge events using (a) best-fit log(C)–log(Q) slopes, (b) changes (% per year) in flow-normalized concentrations, and (c) changes (% per year) in flow-normalized fluxes.

The second part of phase 4 involved accounting for variability in solute concentrations not explained by instantaneous discharge. We tested the effects of hydrologic variables representing basin antecedent soil moisture “wetness”, seasonal stream flows, seasonal to extended dry conditions, and past stream flow events on C–Q relationships. Seasonal flow effects were analyzed using relationships between solute WRTDS concentration–discharge relationships and flow rating curves computed from average daily flows for the water-year, fall and spring seasons. The effects of baseflow, basin wetness (water yield), previous stream flow events, and extended periods of low/no flow conditions on (C–Q) WRTDS residuals were tested with the non-parametric Mann-Kendall trend analysis using the R package ‘Kendall’ (McLeod 2015).

The Mann–Kendall Tau values indicate the strength and direction of monotonic trends, with −1 and 1 representing perfectly negative and positive monotonic trends, respectively. The *p* value was used to assess their significance. The Mann–Kendall test requires that the dependent variable response is monotonic in relation to the independent variable. The strength of the correlation between solute C–Q residuals and hydrologic variables was determined using Kendall’s Tau and the relationship was quantified using the Kendall-Theil robust line (Helsel and Hirsch 2002). The robust line describing the response of solute residuals to hydrologic variables was defined as

1 $$ {\mathrm{Residual}}_{\mathrm{i}}={\beta}_0+{\beta}_1\ast {\mathrm{Hydrologic}\ \mathrm{variable}}_{\mathrm{i}} $$

where Residual_i_ is the residual for day i, ${\mathrm{Hydrologic}\ \mathrm{variable}}_{\mathrm{i}}$ is the hydrologic variables tested on day i, and ${\beta}_0\ \mathrm{and}\ {\beta}_1$are the fitted coefficients for the intercept and slope, respectively.

The fifth and final phase focused on the relationships between nitrate concentrations and hydrologic and land use variables to gain insight into the controlling influences for relationships between nitrate concentrations and flow conditions. The effects of crop areas, agricultural water used, irrigation and conservation practices, rainfall, and evaporative demands on (NO_3_-Q) WRTDS residuals were tested using Mann-Kendall trend analysis.

## Results and discussion

### Concentration–discharge rating curves

**Fig. 5:**
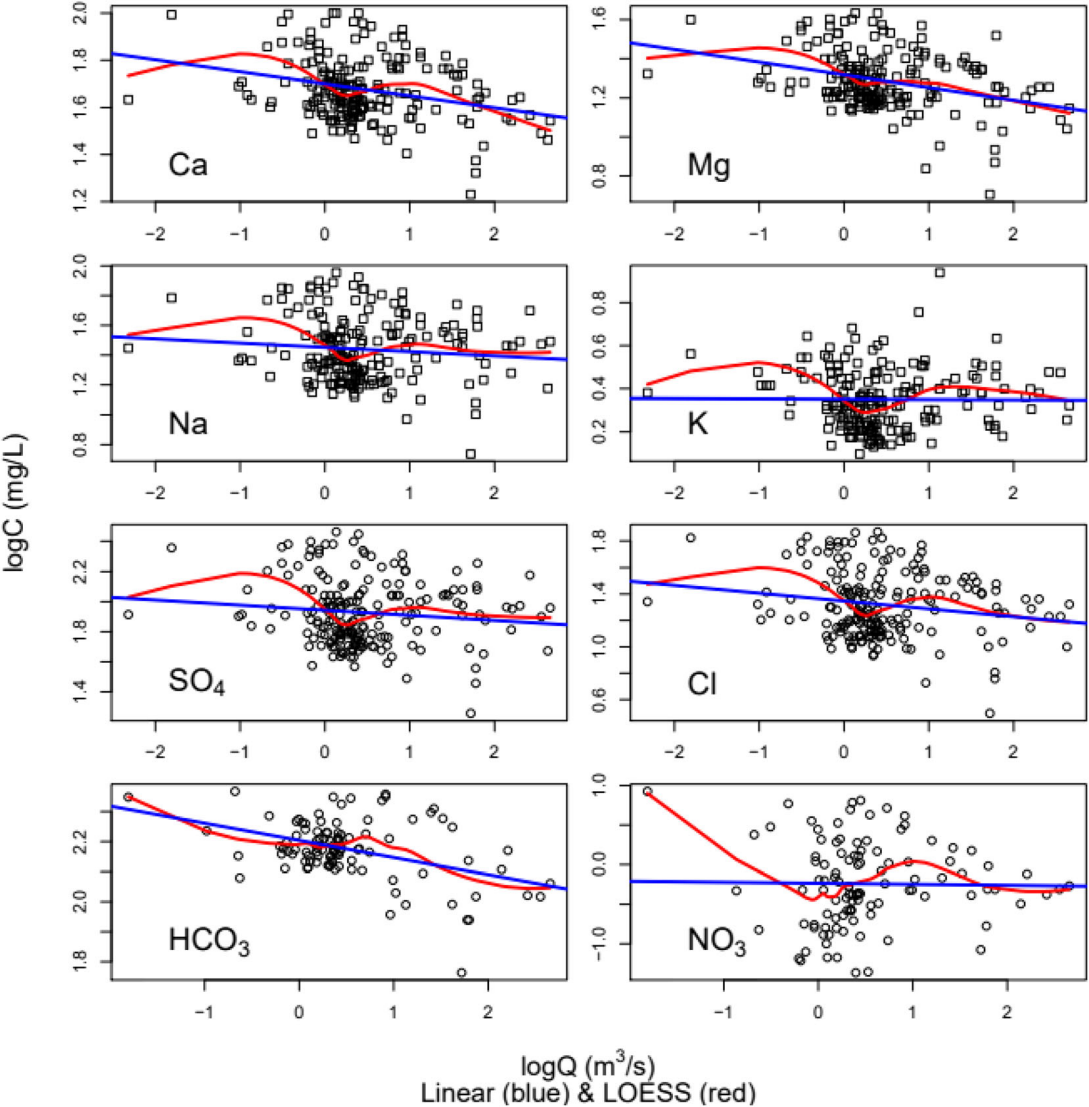
Linear regression and LOESS rating curve models for solute concentration–water discharge behavior in log-log space for over the sampling period

As described above, concentration–discharge relationships have been widely used to estimate mass transport of discharge-dependent constituent loadings. Linear and LOESS regression techniques were applied as single curves fitted to data for the entire temporal domain (1977–2013). A log-linear concentration rating curve describes the C–Q relationship as

2 $$ \log C=\mathrm{blog}(Q)+c+\varepsilon $$

where *c* is the offset of the linear curve, *b* is the slope, and *ε* is the error function. To test the performance of the log-liner curve fit, LOESS rating curves for log *C*–log *Q* relationship were also computed. Table 1 lists model parameter results for both models. While the root means square error (RMSE) statistic is the same, bias estimates differ such that they are slightly lower for the LOESS regression (Table 2). Moreover, LOESS regression residuals were normally distributed for Ca, Mg, SO_4_, and TDS. Figure 5 shows that log-linear curves do not account for the curvature in the C–Q relationships at low and high Q values.

**Table 1:** Solute rating curves

Linear and LOESS regression rating curves					Shapiro-Wilk normality test	
Species	Model	Model equation	*R*^2^	RMSE	*W*	*p* value^a^
Ca	Linear	log Ca = −0.05 logQ + 1.70	0.08	0.13	0.98	**
	LOESS	-	-	0.13	0.99	Normal
Mg	Linear	log Mg = −0.06 logQ +1.32	0.11	0.14	0.97	***
	LOESS	-		0.14	0.98	Normal
Na	Linear	log Na = −0.03 logQ + 1.45	0.01	0.22	0.97	***
	LOESS	-		0.21	0.99	Normal
K	Linear	log K = −0.002 logQ + 0.35	0.00	0.14	0.96	***
	LOESS	-		0.13	0.96	***
SO_4_	Linear	log SO_4_ = −0.03 logQ + 1.94	0.01	0.22	0.98	**
	LOESS	-		0.21	0.99	Normal
Cl	Linear	log Cl = −0.06 logQ + 1.35	0.03	0.25	0.98	**
	LOESS	-		0.24	0.99	Normal
HCO_3_	Linear	log HCO_3_ = −0.06 logQ + 2.20	0.18	0.07	0.97	*
	LOESS	-		0.06	0.97	**
NO_3_	Linear	log NO_3_ = −0.013 logQ − 0.23	0.00	0.39	0.99	*
	LOESS	-		0.37	0.99	Normal

^a^Shapiro–Wilk test result *p* value ranges: normal ≥ 0.5; 0

***0.001, **0.01, *0.05

**Table 2:** Flux bias statistics for the linear, LOESS, and WRTDS model estimates

Constituent	Linear	LOESS	WRDTS
Ca	0.03	0.02	0.01
Mg	−0.02	−0.04	0.01
Na	−0.17	−0.09	−0.01
K	−0.08	−0.03	0.04
SO_4_	−0.17	−0.09	0.01
Cl	−0.11	−0.09	−0.02
HCO_3_	0.00	−0.02	0.03
NO_3_	−0.17	−0.19	0.13

**Fig. 6:**
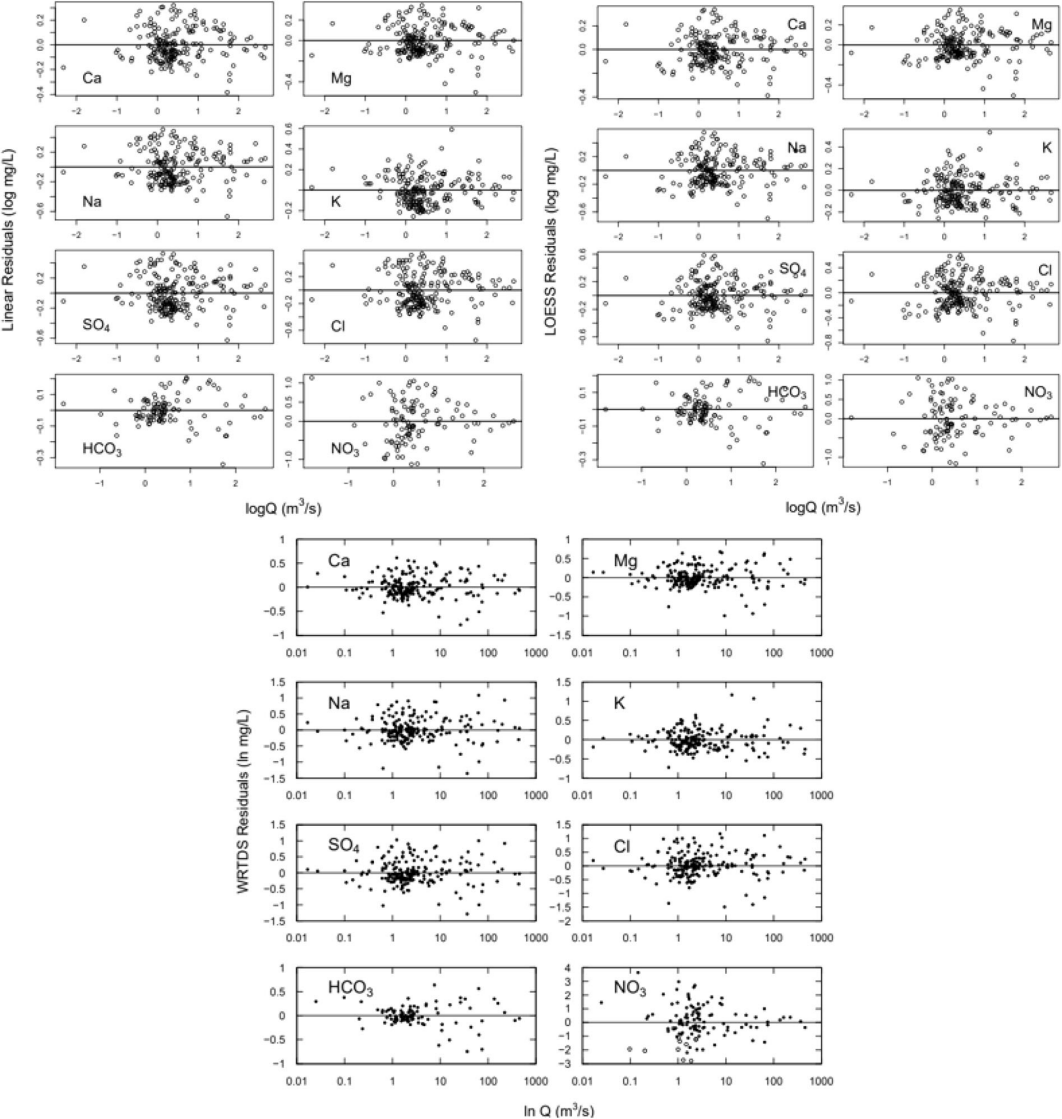
LOESS, linear, and WRTDS residuals

Figure 6 shows residuals for the LOESS, linear, and WRTDS rating curve models. The linear and LOESS C–Q residuals for K and NO_3_ exhibit a randomized structure, whereas for the other species, the structure of residuals exhibits a curvature. WTRDS C–Q residuals for all constituents exhibit no apparent lack of symmetry or curvature. However, for Ca, Mg, Na, K, SO_4_, Cl, and HCO_3_ concentrations, variability of the C–Q residuals is greater at higher discharges and for NO_3_ concentration is greater at lower discharges.

We determined the flux bias statistics from the estimated concentrations from the three models using the following equation:

4 $$ B=\frac{\left(\sum \limits_{\mathrm{i}=1}^{\mathrm{n}}k\times {c}_{\mathrm{i}}^{\hbox{'}}\times {Q}_{\mathrm{i}}\right)-\left(\sum \limits_{\mathrm{i}=1}^{\mathrm{n}}k\times {c}_{\mathrm{i}}.\times {Q}_{\mathrm{i}}\right)}{\left(\sum \limits_{\mathrm{i}=1}^{\mathrm{n}}k\times {c}_{\mathrm{i}}^{\hbox{'}}\times {Q}_{\mathrm{i}}\right)} $$

where the conversion *k*=86.4, ${c}_{\mathrm{i}}^{\hbox{'}}$ is the estimated concentration, *c*_i_ is the observed concentration (mg/L), *Q*_i_ is the discharge on the i^th^ sampling day. Bias values listed in Table 2 suggest that the WRTDS model had a positive bias for NO_3_ and are nearly unbiased for all other constituents (B near zero). We thus used the WRTDS curve residuals (the difference between observed and fitted values) to identify periods of high or low solute concentrations and WRTDS solute concentration estimates to describe changes in solute concentration behavior over time.

### Regime shifts in solute concentrations: inter-annual variability

**Fig. 7:**
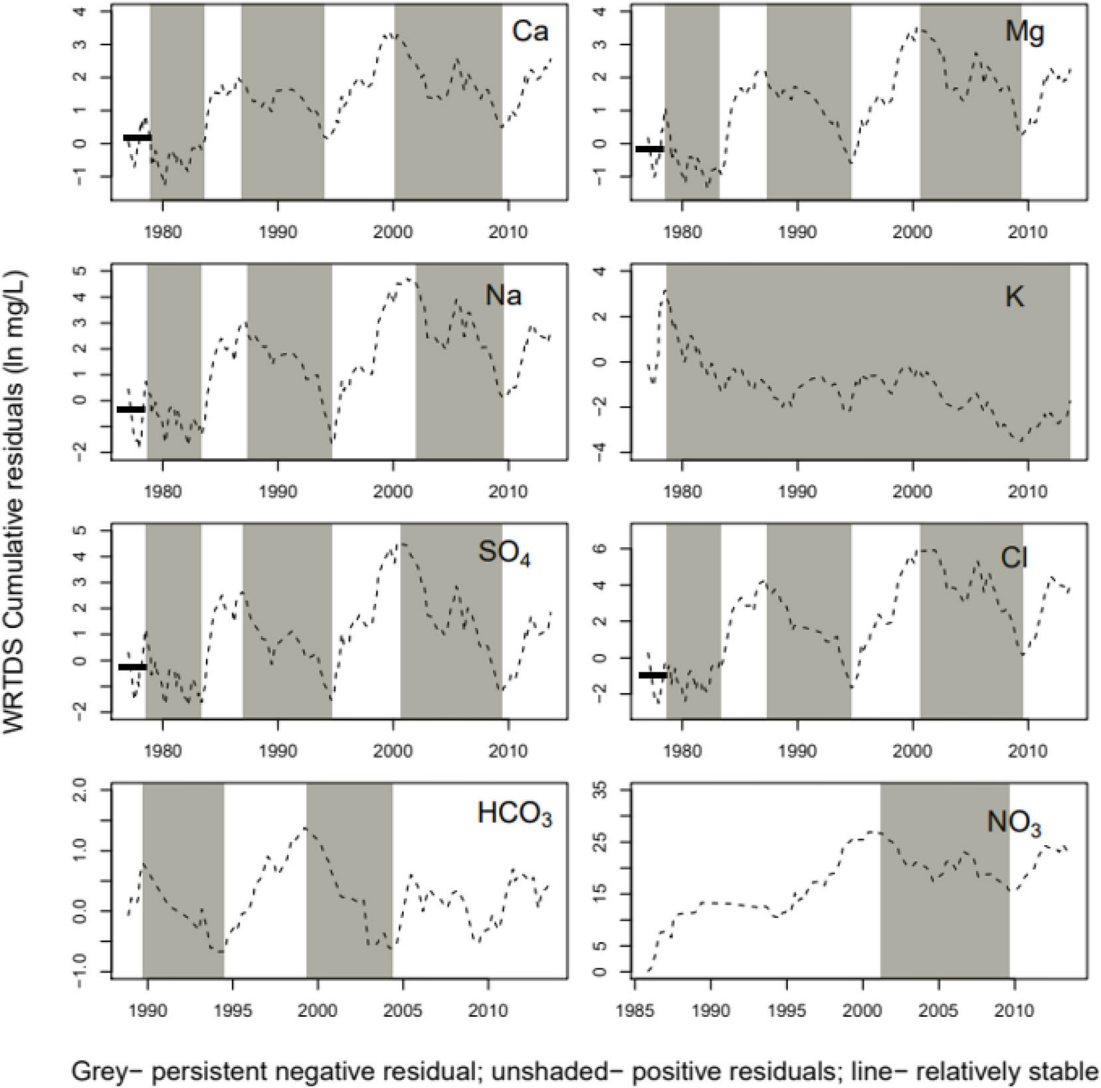
WRTDS residuals sequentially summed over time

Rating curve residuals reveal systematic departures in sample C behavior from that of the rating curve model that is assumed to capture longer-term average behavior. WRTDS residuals are the portion of the concentration signal that is not accounted for by contemporaneous discharge, season, or long-term trends. Thus, positive residuals indicate higher-than-anticipated observed concentrations, while negative residuals indicate a lower-than anticipated observed concentration. Persistent patterns in solute behavior were identified by sequentially summing or accumulating C–Q residuals over time (Hurst 1957). Periods of persistent positive or negative behavior were identified based on the local slope of the cumulative residual curve. Based on the local slope of sequentially summed C–Q residuals, we obtained temporal zones or regimes of higher or lower than expected solute concentration behavior (Fig. 7).

Although the temporal ranges associated with higher or lower than expected solute concentrations differ for each species, there are expected overlaps. Trends in the major cation species Ca, Mg, Na, SO_4_, and Cl affecting ion balances are generally similar such that for these, the temporal zones of persistent negative residuals were between 1978 and 1983, 1986 and 1994, and 2001 and 2008. K residuals were persistently negative from 1978 to 2013. HCO_3_ residuals exhibited persistent negative patterns between 1989–1994 and 2000–2004, and for NO_3_, persistent negative residuals were between 2001 and 2008. Later, using time periods delineated from cumulative C–Q WRTDS residuals, we examine the hydrogeochemical processes occurring in these periods and then the load dynamics associated with discharge variability. This analysis enabled assessment of trends in solute concentration and how controls on stream water concentrations have changed with time.

#### Hydrogeochemical processes controlling solute concentrations over time

The chemical composition of river water is primarily dependent on the geology as well as the geochemical processes and anthropogenic activities that take place within the watershed. Following rainfall events, subsequent dissolution, rock weathering, drainage water, evaporation, and aeration alter surface and soil-water composition. We used compositional relations among major dissolved species and ionic ratio approaches to gain insight into the possible origin of water quality in the watershed (Garrels and Mackenzie 1967; Hounslow 1995). During rock weathering and dissolution, Na, Ca, Mg, SO_4_, HCO_3_, and SiO_2_ ions are added to the water at varying concentrations depending on the leaching rates and rock mineralogy encountered. Temporal variability on chemical concentrations and solute mass balance in the watershed is a result of controls affecting the biogeochemical cycles including hydro-climatic, antecedent hydrological conditions, and anthropogenic forcing including applied fertilizers, soil amendments, and land uses.

**Fig. 8:**
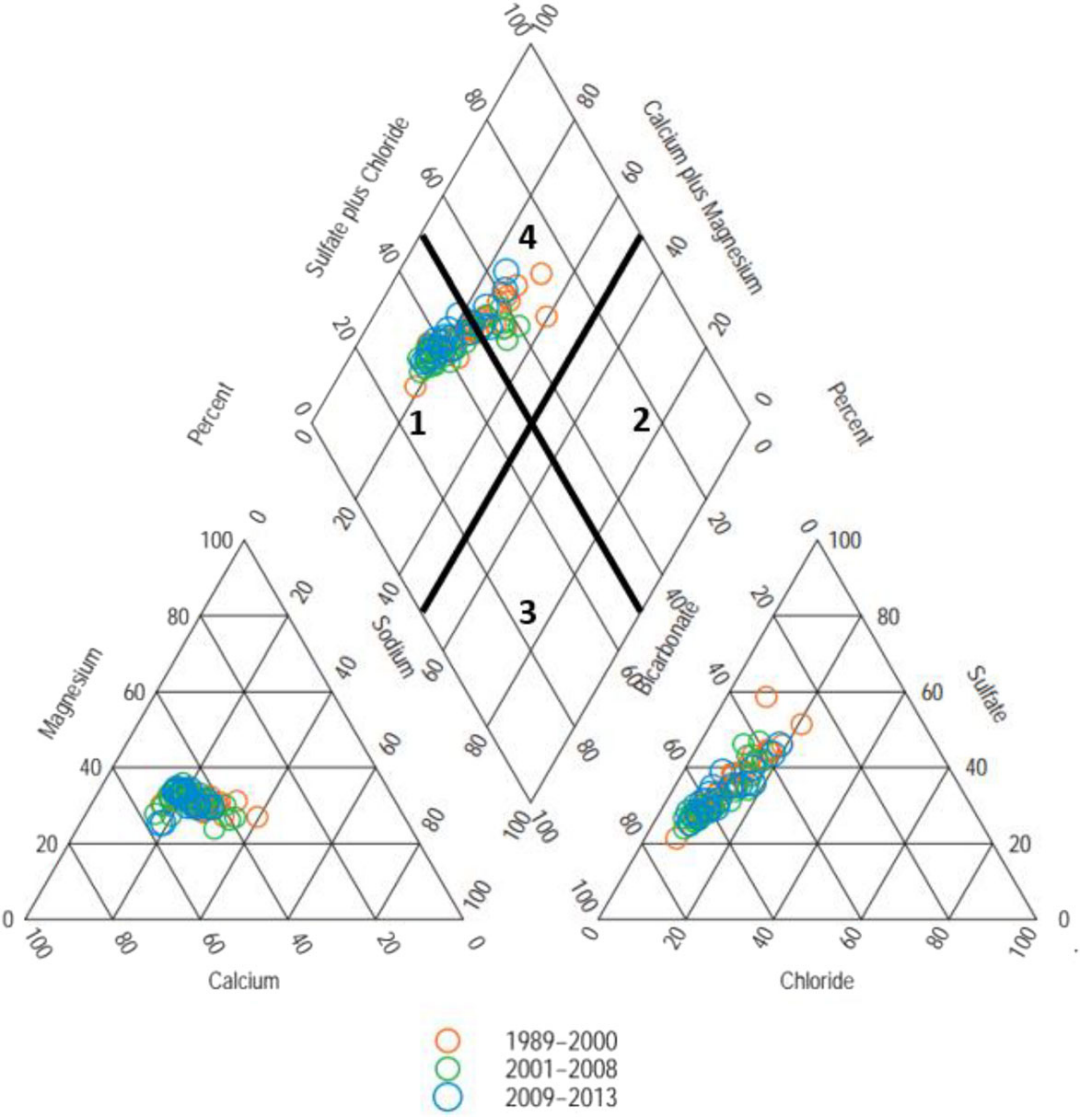
Piper plot of Salinas River (1989–2013) daily sampled water quality, water types categorized as (1) CaMg-HCO_3_, (2) Na+K-Cl or Na+K-SO_4_, (3) Na+K-HCO_3_, and (4) CaMg-Cl or CaMg-SO_4_

Water types as defined by Piper diagram (Piper 1944) are often used in the characterization of waters as a diagnostic tool. Here, most of the Salinas river water samples were Ca-Mg-HCO_3_ and Ca-Mg-Cl/SO_4_ type (Fig. 8). Surface water dissolved species presented in the order of Ca>Mg>Na>K for cations and HCO_3_>SO_4_>Cl>NO_3_ for anions. The mixed Ca-Mg-HCO_3_ water type suggests mineral dissolution with sufficient recharge from freshwater and Ca-Mg-Cl or SO_4_ and Na-K-Cl/SO_4_ types suggest mixing of freshwater with water from contaminated sources (Ako et al. 2012). Ca+Mg dominates Na+K and HCO_3_ exceeds SO_4_+Cl at all time periods. The high Ca, Mg, and HCO_3_ concentrations suggest that chemical weathering processes occurred in the watershed.

We used the temporal periods defined by NO_3_ residual patterns (1989–2000, 2001–2008, and 2009–2013) to track changes in the surface water chemistry and the likely sources of change. Nitrate concentrations ranged from 184 to 647, 92 to 561, and 145 to 660 mg/L and pH ranges were 7.7–8.9, 7.7–8.5, and 8.0–8.6 for the periods 1989–200, 2001–2008, and 2009–2013, respectively. HCO_3_ was the dominant anion in the lower Salinas River and varied from a minimum of 58 mg/L to a maximum of 233 mg/L. Naturally, rainwater is slightly acidic from reaction with carbon dioxide in the atmosphere (Krauskopf and Bird 1994).

**Fig. 9:**
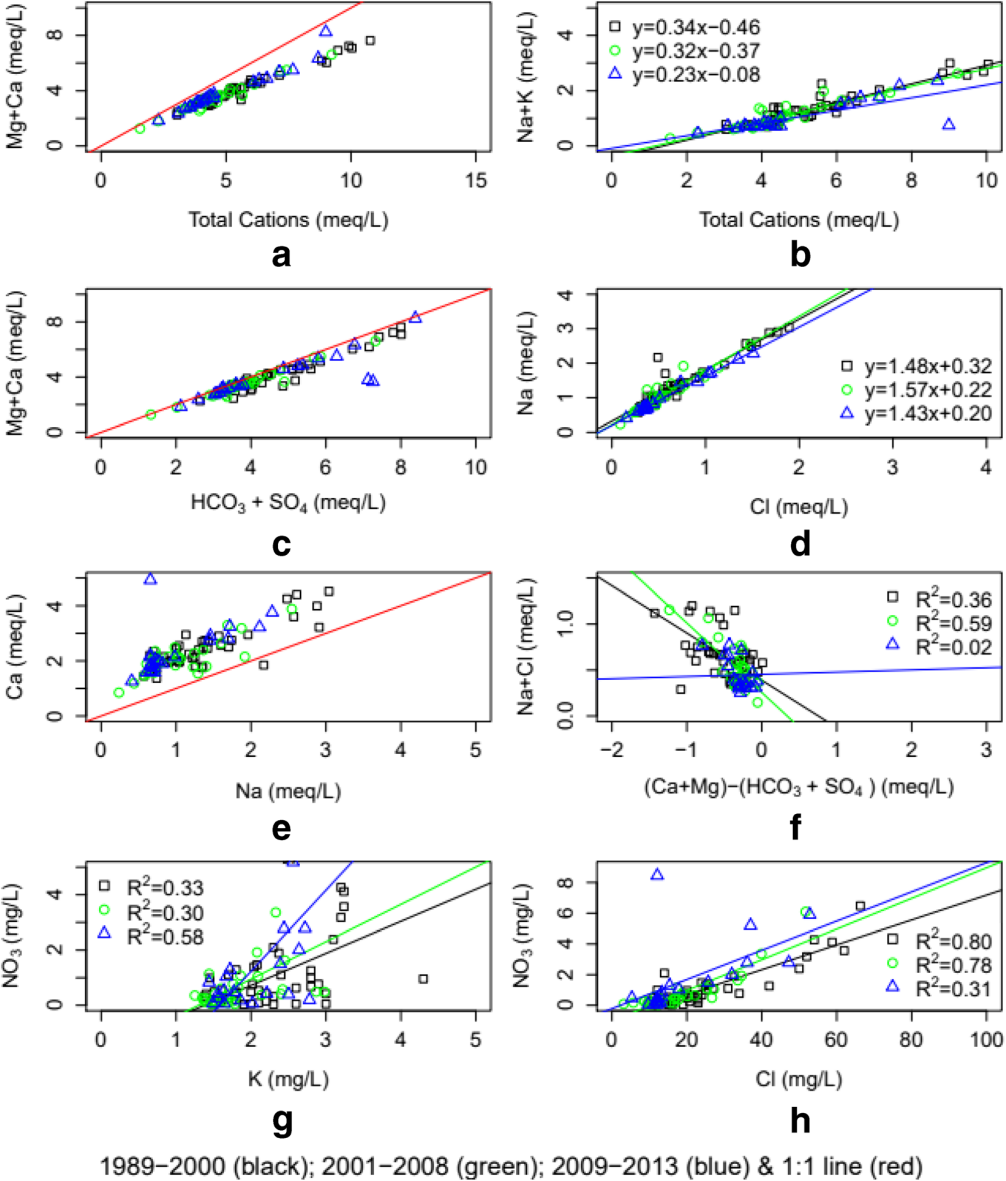
Interrelationship among dissolved species

The plot (Fig. 9a) of Ca+Mg vs. total cation concentrations was below the 1:1 line suggesting that weathering reactions are controlling major cation concentrations. The equivalent ratio of Na+K/total cations was less than 0.5 suggesting that silicate weathering was the source of major cations (Fig. 9b). Moreover, the clustering of points on the Ca+Mg vs HCO_3_+SO_4_ plot near the 1:1 line (Fig. 9c) indicates that in addition to silicate weathering, basic dissolution of carbonate minerals may also contribute to Ca and Mg enrichments in water (Hounslow 1995). Further support for weathering is provided by the strong correlations (Table 3) observed between TDS with Ca, Mg, Na, and HCO_3_ concentrations (Tau=0.78, 0.79, 0.76, and 0.55, respectively). In addition, the significant positive correlations of SO_4_ with HCO_3_, Ca, Mg, and K concentrations suggest that weathering was the main source of SO_4_ (Tau=0.44, 0.67, 0.67, and 0.65, respectively) in the surface water.

**Table 3:** Kendall correlation coefficients for dissolved species

	pH	Ca	Mg	Na	K	SO_4_	Cl	HCO_3_	NO_3_	SiO_2_	TDS
pH	*1.00*										
Ca	0.12	*1.00*									
Mg	*0.19*	*0.80*	*1.00*								
Na	0.01	*0.55*	*0.62*	*1.00*							
K	−0.11	*0.55*	*0.48*	*0.50*	*1.00*						
SO_4_	0.02	*0.67*	*0.67*	*0.73*	*0.65*	*1.00*					
Cl	0.03	*0.60*	*0.66*	*0.82*	*0.49*	*0.70*	*1.00*				
HCO_3_	*0.15*	*0.72*	*0.66*	*0.35*	*0.36*	*0.44*	*0.41*	*1.00*			
NO_3_	−0.01	*0.44*	*0.46*	*0.54*	*0.36*	*0.46*	*0.57*	*0.30*	*1.00*		
SiO_2_	*−0.15*	*0.20*	*0.20*	*0.45*	*0.25*	*0.28*	*0.43*	0.12	*0.46*	*1.00*	
TDS	0.06	*0.78*	*0.79*	*0.76*	*0.61*	*0.82*	*0.76*	*0.55*	*0.52*	*0.32*	*1.00*

Italicized values indicate significant correlations at *p*< 0.05

The Na/Cl ratio is greater than that of seawater 0.86 (Fig. 9d) indicating that simple precipitation was not the only process responsible for the Na ions present. Dissolution of sodium feldspar was likely one of the processes involved (Fisher et al. 1997). The plot of Ca vs. Na was above the 1:1 line (Fig. 9e) indicating that ion exchange between Ca and Na was taking place in addition to Na from feldspar dissolution. The data plotted in Fig. 9f verifies the significance of ion exchange as the relationship between (Na+Cl) vs. (Ca+Mg)-(HCO_3_+SO_4_). Fisher et al.  (1997) found that a linear relationship between (Na+Cl) vs. (Ca+Mg)-(HCO_3_+SO_4_) with a slope of −1.0 suggest that ion exchange is a significant geochemical process controlling water chemistry. Slopes for the periods between 1989–2000, 2001–2008, and 2009–2013 were −0.51, −0.76, and +0.02, respectively, indicating that ion exchange in the latter period was not taking place in the system.

The presence of NO_3_ and Cl in the surface water sampling appears to be associated with human activities. We tested correlations between NO_3_ with Cl and K for the different periods (Fig. 9 g and h). The positive correlations between NO_3_ and Cl support the conclusion that inputs of NO_3_ and Cl are human-related activities—*R*^2^=0.31 to 0.80. We found a positive relationship between NO_3_ and K indicating that agricultural practices are the major source of NO_3_ in the area (Fig. 9h). This correlation is much more significant during the 2009–2013 period indicating greater agricultural impacts associated with NO_3_ loading to the river then. Furthermore, Smith et al. (2018) noted that increased NO_3_ concentrations were associated with increased nitrification processes in the watershed due to N-fertilizer inputs.

#### Assessment of the effects of stream flow on shifts in river solute concentrations

River solute concentrations depend on a wide variety of factors including hydrologic characteristics of the watershed, seasonal influences, biological activity, and geochemical reactions. Using the temporal periods defined by NO_3_ C–Q residual patterns (1989–2000, 2001–2008, and 2009–2013), we tracked changes in correlations between solute concentrations and discharge. Flows in the lower Salinas River are controlled by releases from Nacimiento and San Antonio dams that are used to enhance recharge to the unconfined aquifer during dry months of the year and to maintain continuous flow in the streambed from Bradley to Chualar. Further, in 2010, the Salinas River Diversion Facility began delivering reservoir water for irrigation purposes. Despite streamflow regulation, daily flows have varied from year to year. We explicitly consider the role of seasonal flow, basin antecedent moisture “wetness”, and baseflow on changes in river solute concentrations over time.

Several authors (Hirsch et al. 1982; Hunt and Foster 1985; Johnson 1979; Galat 1990) developed models that describe correlations between solute concentrations and discharge. Typically, the type I relationship is associated with dilution, that is, solute concentration decreases as discharge increases. This type I arises when point source discharge is relatively constant and independent of river flow or the case when high concentration baseflow is diluted by a lower concentration surface flow. A type II relationship characterizes a flow-driven release in which concentration increases as a function of discharge, such as when loading is from non-point sources or when precipitation increases runoff and washes solutes from the watershed into the river (wash off). Type III relationships combine types I and II such that point sources dominate at low flows producing a dilution effect while at higher discharges, non-point sources become significant and loading dominates. We add a type IV relationship that is characterized by constant concentrations over widely varying discharges (chemostatic behavior). Godsey et al. (2009) found that weathering-derived solute (Ca, Mg, SiO_2_, Na) concentrations were relatively constant despite large variations in streamflow across 59 pristine US watersheds and defined chemostatic response as when *b* ≈ 0 in the classic power relationship *C* = aQ^b^. Basu et al. (2010) suggested that the solute export pattern from agricultural basins was characterized by near constant flow-weighted concentration at annual time scales. S. E. Thompson et al. (2011) hypothesized that the relative stability of concentration under widely varying discharge (chemostatic responses) emerges because of increased solute mass stored in a catchment. Where these stores are not intrinsic, they should arise following persistent external inputs to the catchment.

Italicized values indicate significant correlations at *p*< 0.05

We used the slope of the log(*C*)–log(*Q*) regressions within the differentiated temporal periods 1989–2000, 2001–2008, and 2009–2013 to consider load dynamics from discharge variability. Parameter information about the best-fit log(*C*)–log(*Q*) slopes for each solute within each period are summarized in Table 4 along with the correlation coefficients and significance statistic for the slope. *R*^2^ values are very low especially as *b* approaches zero. It was therefore difficult based on this model to conclude the underlying processes that control behavior of solute concentrations over time. However, significant *p* values for slopes (b) resulting from the regression were found for HCO_3_ concentrations between 1989–2000 and 2001–2008. The slope (b) was negative indicating dilution effects such that concentration decreased with increasing discharge. Negative and significant slopes were also found for Mg concentrations between 2001 and 2013. A positive and significant slope was found for K concentrations between 2001 and 2008 indicating that concentrations increased with discharge.

**Table 4:** Changes in solute behavior with time for different periods based on cumulative NO_3_ residuals

Species	Time period	log *C* = b* log *Q* + *c*			WRTDS results	
		*B*	*b p* value^a^	*R*^2^	Change in concentration (% per year)	Change in flux (% per year)
Ca	1989–2000	−0.03	ns	0.04	*−0.4*	*−0.5*
Mg		−0.04	ns	0.06	*−0.5*	*−0.5*
Na		0.04	ns	0.03	−0.9	0.6
K		0.02	ns	0.02	−0.6	−0.4
SO_4_		0.04	ns	0.02	−0.7	0.0
Cl		0.01	ns	0.001	−0.6	0.1
HCO_3_		−0.04	**	0.14	−0.9	−0.5
NO_3_		0.2	ns	0.07	1.7	−1.0
Ca	2001–2008	−0.06	ns	0.09	0.2	−0.6
Mg		−0.1	*	0.18	−0.1	−1.2
Na		0.02	ns	0.004	−1.0	−2.2
K		0.09	**	0.3	0.1	−0.2
SO_4_		0.02	ns	0.006	−0.2	−2.4
Cl		−0.04	ns	0.01	−0.4	−2.0
HCO_3_		−0.1	**	0.3	*−0.1*	*0.0*
NO_3_		0.02	ns	0.001	4.5	0.3
Ca	2009–2013	−0.07	ns	0.13	*−1.0*	*−0.9*
Mg		−0.1	*	0.19	*−1.0*	*−1.1*
Na		0.05	ns	0.03	−1.8	−1.4
K		−0.03	ns	0.04	*−0.8*	*−0.7*
SO_4_		−0.11	ns	0.08	−2.1	−1.7
Cl		0.05	ns	0.02	−1.7	−1.9
HCO_3_		−0.05	ns	0.11	0.0	0.2
NO_3_		−0.02	ns	0.001	4.8	2.4

***0.001, **0.01, *0.05

Chemostatic solute behavior (type IV)

Italicized values represent chemostatic solute behavior (Type IV)

^a^*p* value ranges: ns > 0.5; 0

The slopes of the log(*C*)–log (*Q*) regressions were rather inconclusive for most of the solutes. As such, we used estimates of concentration and flux (specifically flow normalized concentration and flux) from the WRTDS model to further describe changes in solute concentrations–discharge relationship. Annual average flow-normalized concentration is more indicative of trends associated with low to moderate stream flows, while annual average flow normalized flux is more indicative of trends associated with high stream flows. We first describe the computation of flow-normalized concentration and flow-normalized flux in the WRTDS model and use the changes (in % per year) to infer trends in solute concentrations with stream flow ranges.

In addition to the computation of daily concentration estimates, the WRTDS model computes daily estimates of flux (kg/day) as

3 $$ \mathrm{Flux}={C}_{\mathrm{pred}}\ast Q\ast 86.4 $$

where *C*_pred_ is the daily estimate of concentration (mg/L; *Q* daily mean discharge (m^3^/s)) and 86.4 the unit conversion. In general, annual average concentrations will tend to reflect conditions over the many days of low to moderate stream flow, and these are strongly determined by point sources and base-flow contributions. Conversely, annual average load values tend to reflect the relatively few days of very high stream flow each year, and thus, they are strongly determined by nonpoint-source runoff-related contributions.

In the WRTDS model, flow-normalization estimates of concentrations and fluxes for the entire period remove the random variations in these quantities that arise from the random variations in discharge (Hirsch et al. 2010; Medalie et al. 2012). The flow-normalization procedure eliminates the influence of temporal discharge patterns by using the probability distribution function (pdf) of discharge values for that day. The flow-normalized concentration on a specific day (a specific value of *T*) is calculated as

4 $$ \mathrm{E}\left[\mathrm{C}\left(\mathrm{T}\right)\right]=\int \mathrm{w}\left(\mathrm{Q},\mathrm{T}\right)\ast {\mathrm{f}}_{\mathrm{T}}\ \mathrm{dQ} $$

where E[C(T)] is the flow-normalized concentration for time T, w(Q, T) estimates of concentration as a function of Q and time T and f_T_ is the pdf of discharge specific to a particular date. Since flow-normalization values remove large variability from the annual averages they are suitable for evaluating long-term trends (Hirsch et al. 2010).

The premise of flow-normalization is that the discharge measured on a specific date, say 1 April 1979, was a random occurrence from the probability distribution of discharges that were measured on every April 1st over the period of record (here, 1977–2013). To estimate the flow-normalized concentration for 1 April 1979, the regression model that estimates concentrations (Equation (2)) is run 27 times, where the time variable is always fixed at 1 April 1979 and the discharge variables are the 27 April 1st discharges in the record. The flow-normalized concentration for 1 April 1979 is the mean of the estimated concentrations generated from the 27 model runs. This process is repeated for every date in the 27-year record to generate the daily histories of flow-normalized concentration and flux.

Flow normalization allows observation of changes in the way the watershed responds to the full range of hydrologic conditions and not just a temporal pattern of hydrologic conditions that happen to occur in the period of record. We computed changes (in % per year) in flow-normalized concentrations and fluxes for the time periods 1989–2000, 2001–2008, and 2009–2013 for all species (Table 4). Flow normalized flux reflects changes in solute concentrations associated with high stream flow contributions while flow normalized concentrations reflect changes in solute concentrations associated with low stream flow contributions.

We found that in the period from 1989 to 2000 (dry period) concentrations, contributions of low to high stream flows decreased for Ca, Mg, K, and HCO_3_. In the same period, concentrations associated with low stream flows increased for NO_3_ and concentration contributions of high stream flows increased Na and Cl concentrations. During the wet period between 2001 and 2008, concentrations associated with high stream flows decreased for all solutes except NO_3_ that had increased concentrations along with Ca and K. The dry period from 2009 to 2013 concentration contributions associated with low and high stream flows decreased for all solutes; however, NO_3_ concentrations increased. Changes in flow-normalized concentration and flux were equal in some instances, for instance, Ca and Mg concentrations between 1989–2000 and 2009–2013 and HCO_3_ concentrations during 2001–2008 and K concentrations during 2009–2013 (highlighted in Table 4). The equality indicates that trends in the log of concentration were the same across the full range of discharges and the full range of seasons, that is, chemostatic solute behavior.

**Fig. 10:**
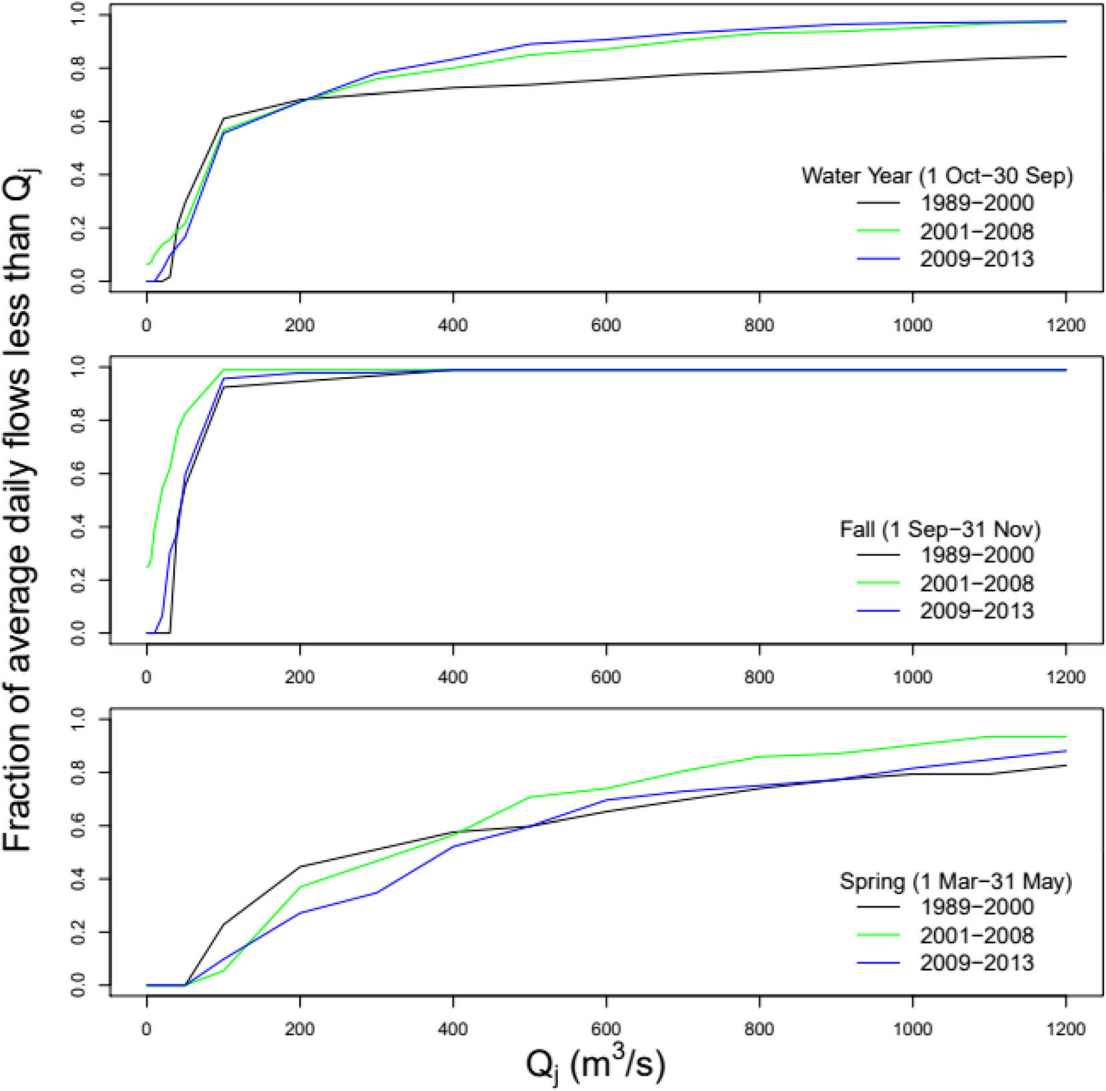
Average daily flow rating curves for the periods 1989–2000, 2001–2008, and 2009–2013

Seasonal effects can have a significant influence on river solute exports. We constructed water year, fall and spring season flow rating curves from average daily flows for the periods 1989–2000, 2001–2008, and 2009–2013 (Fig. 10). Fractions of flows less than 10 m^3^/s were 0%, 10%, and 0% during 1989–2000, 2001–2008, and 2009–2013 periods, respectively. While flows less than 20 m^3^/s were 0% during 1989–2000, 14% during 2001–2008 and 4% during 2009–2013. Fractions of flows more than 50 m^3^/s were 71% during 1989–2000, 78% during 2001–2008, and 83% during 2009–2013. Mean annual rainfall was 624 mm, 1077 mm, and 636 mm while climate water demand (rainfall-potential evapotranspiration) was 827 mm, 725 mm, and 906 mm for 1989–2000, 2001–2008, and 2009–2013 periods, respectively. Although 1989–2000 and 2009–2013 were similarly dry, the proportion of high stream flows increased in the latter period sustained by dam releases. Dam regulated flows controlled stream flows during 2009 to 2013 particularly releases associated with the Salinas River Diversion Facility that began delivering irrigation water in 2010. We attribute decreases in Ca, Mg, Na, K, SO_4_, and Cl concentrations between 2001 and 2008 to high rainfall and therefore increased runoff resulting in dilution effects. On the other hand, decreases in concentrations of the aforementioned solutes from 2009 to 2013 are likely due to the increases in the proportion of high stream flows maintained by dam releases, though reservoir releases also increased stream HCO_3_ concentrations.

We conducted Mann-Kendall trend analyses comparing daily baseflow and monthly water yield with solute WRTDS C–Q residuals for the different constituents. Baseflow had a significant and positive correlation with Ca, Mg, Na, and Cl concentration residuals. Baseflow thus tended to increase concentrations of these solute species. The regression slope coefficients confirm these results as summarized in Table 5. Basin wetness represented by monthly water yield had a negative and significant correlation with concentration residuals for Mg, Na, K, SO_4_, and Cl; runoff decreased transport of these solute species. Linear regression slope coefficients for the significant Kendall Tau correlations confirm these trends as summarized in Table 5.

**Table 5:** Mann-Kendall trend analysis for basin wetness and baseflow hydrologic variables

Species	Baseflow (daily)			Water yield (monthly)		
	Tau	*p* value^a^	*β*_1_	Tau	*p* value^a^	*β*_1_
Ca	0.15	**	0.00120	−0.09	ns	−0.021
Mg	0.13	**	0.00100	−0.13	**	−0.013
Na	0.13	**	0.00004	−0.13	**	−0.030
K	0.03	ns	−0.00010	−0.10	*	−0.002
SO_4_	0.14	**	−0.00100	−0.12	*	−0.081
Cl	0.18	***	0.00100	−0.10	*	−0.017
HCO_3_	0.09	ns	0.01000	−0.07	ns	−0.028
NO_3_	0.11	ns	0.00004	−0.08	ns	−0.002

^a^Two-sided *p* value ranges: ns > 0.5; 0

***0.001, **0.01, *0.05

For all solute concentrations, dry conditions limited transport of solute concentrations while increasing solute supplies for later transport during wet conditions. Wet conditions had the effect of depleting solute supplies in later periods. Moreover, several dynamic mechanisms controlling concentration–discharge relationships are at play as shown in Table 6. These include event responses as described by the concentration–discharge relationship model types I, II, III, and IV—dilution effects, flow-driven releases, combination of dilution effect at low flows, and flow-driven concentration increases at high flows and chemostatic responses, respectively. Additional controls are the levels of mass solute stores, geochemical processes taking place in the watershed and dam-regulated flows.

**Table 6:**
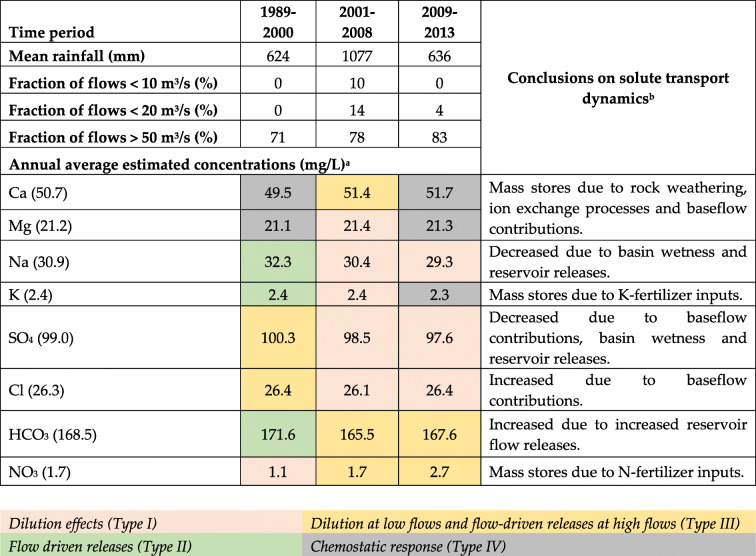
Summary of hydrologic controls on solute transport

^a^Mean concentration of average predicted conditions shown in ()

^b^Wet conditions have an exhaustion effect on all solutes concentrations; dry conditions are transport-limited

Cation exchange during 2009–2013 is likely taking place as shown by the slope of the plot of (Na+Cl) vs. (Ca+Mg)-(HCO_3_+SO_4_) (Fig. 9f). Between 2009 and 2013, concentration–discharge relationships for Ca, Mg, and K exhibit chemostatic behavior, that is concentrations remain constant under varying discharge conditions. Given that Ca and Mg are of geogenic origin, we conclude that the constant C–Q relationship emerges as a result of mass stores of these solutes due to cation exchange, rock weathering processes, and baseflow contributions. On the other hand, the presence of K is associated with human activities particularly K-fertilizer inputs. Concentrations of NO_3_ have increased for all stream flow ranges (low to high) indicating an emergent feature of agricultural land uses in the watershed characterized by in particular the application of N-fertilizers.

We described regime shifts in solute concentrations over multi-decade scales and identified variables that influenced solute behavior over time. Further, we described that hydrologic event history, for example wet conditions preceding dry conditions, influenced transport of all solute concentrations to the lower Salinas River. Next, we explicitly test the strength of the relationships between solutes C–Q WRTDS residuals and antecedent hydrologic conditions. We used hydrologic variables representing seasonal to inter-annual basin aridity and variables accounting for the number of days elapsed since a flow event (stream discharge). These relationships allow us to account for variability in solute concentrations influenced by lasting or memory effects of a previous flow event as well as processes occurring after prolonged dry conditions.

#### Effect of antecedent hydrologic conditions on C–Q residuals

We examined the effects of antecedent hydrologic conditions using the Mann-Kendall trend analysis method. Seasonal and long-term basin aridity was examined through set of variables *Q*_0.1_ calculated as the sum of days when flows were *Q* ≤ 0.1 m^3^/s before sampling. Mean daily flow of 0.1 m^3^/s represented the 25th percentile on the annual duration curve from 1989 to 2013. The *Q*_0.1_ variables sum the number of days before the sampling date that had *Q* ≤ 0. 1 m^3^/s; we tested time windows from 1 to 2000 days. These variables were used to test the effects of prolonged dry conditions on solutes C–Q residuals. Time window of ~10–100 days tested seasonal-scale effects, that is, the effects on the solute C–Q WRTDS residuals of dry conditions lasting from a few days to 3 months. Analyses at longer time frames (>100 days) test the effect of extended low or no flow conditions on C–Q residuals.

Hydrologic event history was represented by the variable *Q*_j_,time. This is a measure of the elapsed time between the last daily mean greater than or equal to *Q* of a given magnitude j and the date of sampling created by *Q*_j_ values from 1 to 1200 m^3^/s. This variable was used to test changes in solute C–Q WRTDS residuals after a particular high or low magnitude flow event. For example, this variable enables testing of how high flow events associated with flooding—an episodic event—affect solute transport long after the event has taken place. Together, the *Q*_0.1_ and *Q*_j_,time variables help better define the specific hydrologic processes that influence solute concentrations after a drought or a specific flow event.

**Fig. 11:**
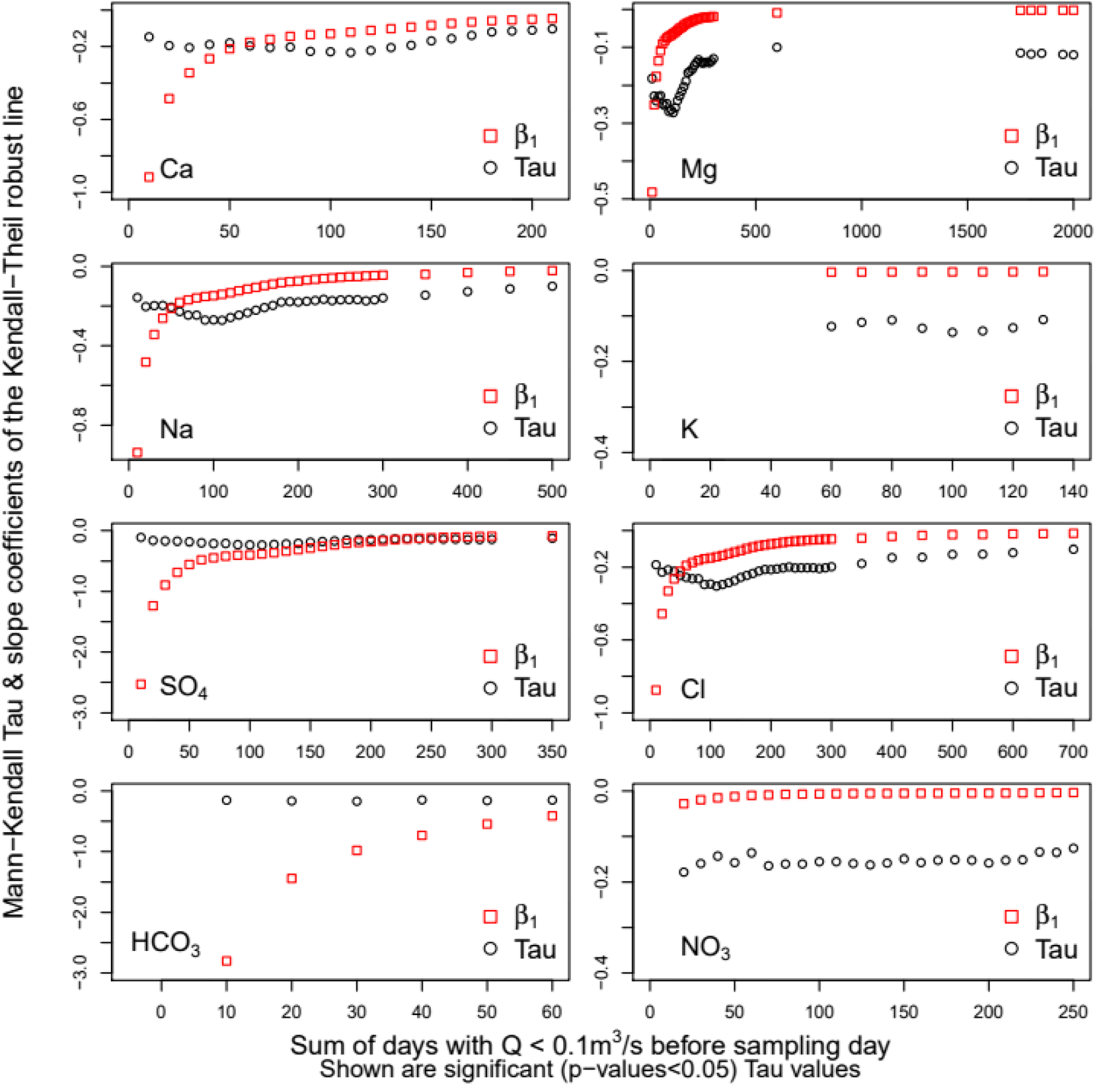
Mann-Kendall analysis of monotonic trends in solute WRTDS residuals in relation to the sum of days with *Q* ≤ 0.1 m^3^/s over summation windows of 10–2000 days and corresponding slope coefficients of the Kendall-Theil robust line

Solute C–Q residuals responded differently to antecedent hydrologic conditions (Table 7). Seasonal to long-term dry conditions limited solute transport to the stream (Fig. 11). Dry conditions had significant and negative correlations with C–Q residuals for all solutes; however, the time scales of significance differed. Dry conditions over seasonal time scales <100 days lowered HCO_3_ concentrations; dry conditions over seasonal to annual time scales decreased concentrations of Ca, K, SO_4_, and NO_3_; and dry conditions over seasonal to inter-annual time scales decreased Mg, Na, and Cl concentrations. The slope coefficient of the Kendall-Theil robust line confirms the trends indicated by the Mann-Kendall tau coefficient. Additionally, we note that the degree to which dry periods reduce solute transport to the river decreases with the length of the dry period as slopes increased over the summation window (Fig. 11). Possibly, a case where the effect of aridity allowed for storage and accumulation of salts in the soil and in dams due to evapo-concentration. Over time, however, these salts were slowly released through seepage and dam releases into the river. This indicates that the ability of dry conditions to suppress salt transport to the river diminished with time.

**Table 7:** Mann-Kendall trend analysis, effects of antecedent hydrologic conditions

Solute species	Maximum days of transport limited dry conditions			Low end flushing flows			High end flushing flows		
	∑*Q*_0.1_ (time window)	Tau	Two-sided	*Q*_j_ (m^3^/s)	Tau	Two-sided	*Q*_j_ (m^3^/s)	Tau	Two-sided
			*P* value^a^			*P* value^a^			*P* value^a^
Ca	210	−0.10	*	3	−0.12	*	90	−0.12	*
Mg	2000	−0.12	*	7	−0.10	*	1200	−0.18	**
Na	500	−0.10	*	7	−0.11	*	1200	−0.17	**
K	130	−0.12	*	-	-	-	-	-	-
SO_4_	350	−0.13	*	3	−0.11	*	1000	−0.12	*
Cl	700	−0.10	*	2	−0.11	*	1200	−0.19	***
HCO_3_	60	−0.15	*	-	-	-	-	-	-
NO_3_	240	−0.14	*	7	−0.13	*	1200	−0.14	*

^a^Two-sided *p* value ranges: ns > 0.5; 0

***0.001, **0.01, *0.05

**Fig. 12:**
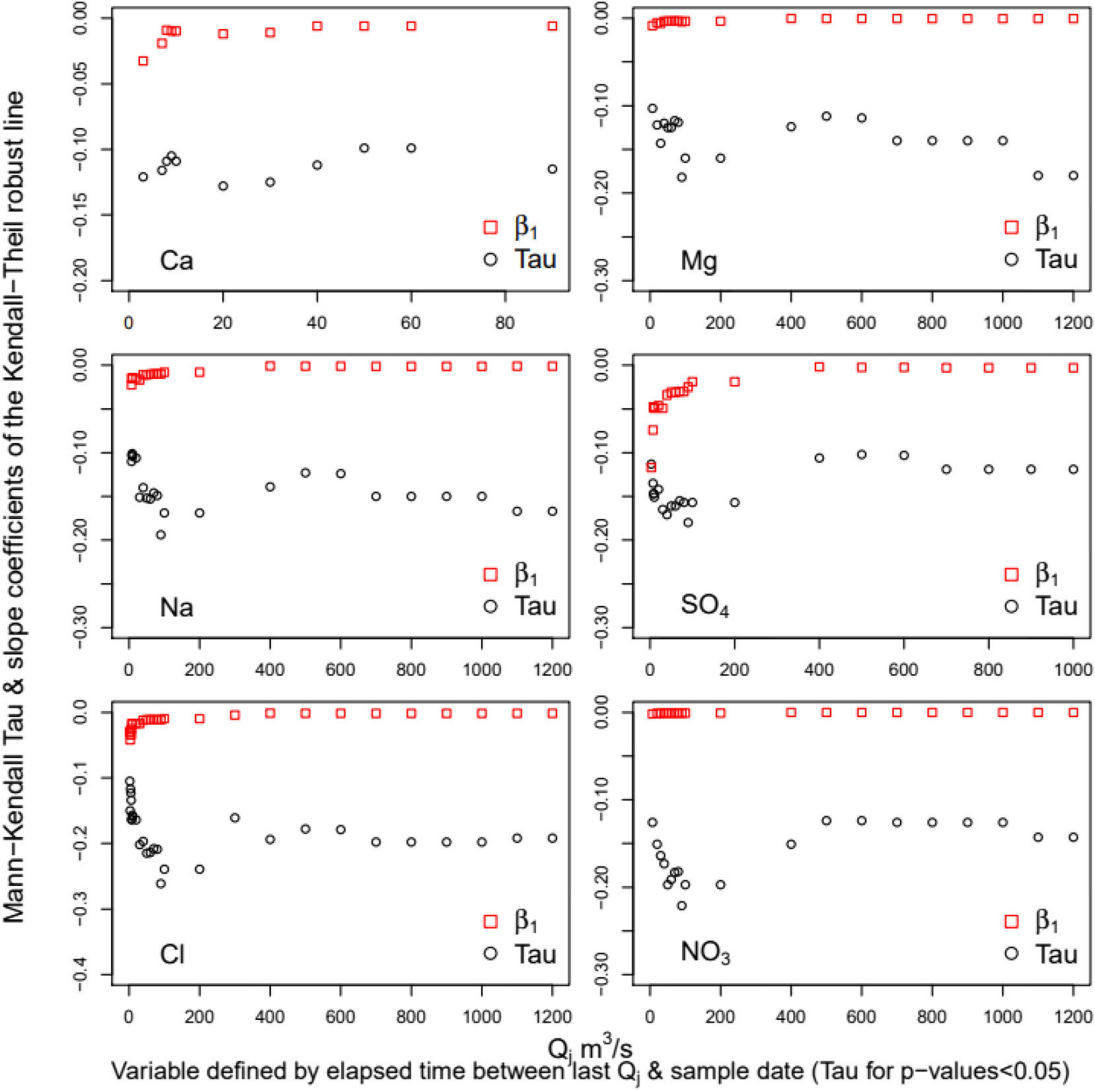
Mann-Kendall analysis of monotonic trends in solute WRTDS residuals in relation to elapsed time since the last streamflow event of magnitude *Q*_j_ and corresponding slope coefficients of the Kendall-Theil robust line

Effects of episodic events on solute transport showed a range of stream flows acted as flushing functions decreasing the concentration supplied to the stream for some time after the event. For example, Ca concentrations decreased with increased elapsed time since the last daily mean flow of 3–90 m^3^/s; Mg, Na, and NO_3_ concentrations decreased for 3–1200 m^3^/s, SO_4_ concentration decreased for 3–1000 m^3^/s, and Cl concentrations decreased for 2–1200 m^3^/s, while K and HCO_3_ concentrations were not affected by episodic storm events (Fig. 12). Based on the magnitude of the slope coefficient of the Kendall-Theil robust line, a streamflow of 3 m^3^/s effectively decreased Ca, SO_4_, and Cl concentrations while 7 m^3^/s effectively decreased Mg, Na, and NO_3_ concentrations in the river for some time after the event.

### Relationship between NO_3_-Q residuals, climate and land use variables

Nitrate concentrations increased with time suggesting an emerging problem associated with agricultural production. However, the history of the nitrate origin is not clear, that is, are the increasing concentrations associated with agricultural production from the past year or past decade or more? Here, we use the record of nitrate residuals to link internal ecosystem controls on the nitrate export (i.e., land management practices). We tracked changes in NO_3_ concentration over time to understand how they respond to external factors such as climate change. Analysis of multivariate records for the watershed include crop areas, agricultural water used, irrigation and conservation systems implemented, rainfall, and potential evapotranspiration. These variables enabled an understanding of how stream nitrate concentrations responded to both internal and external changes within the watershed.

**Fig. 13:**
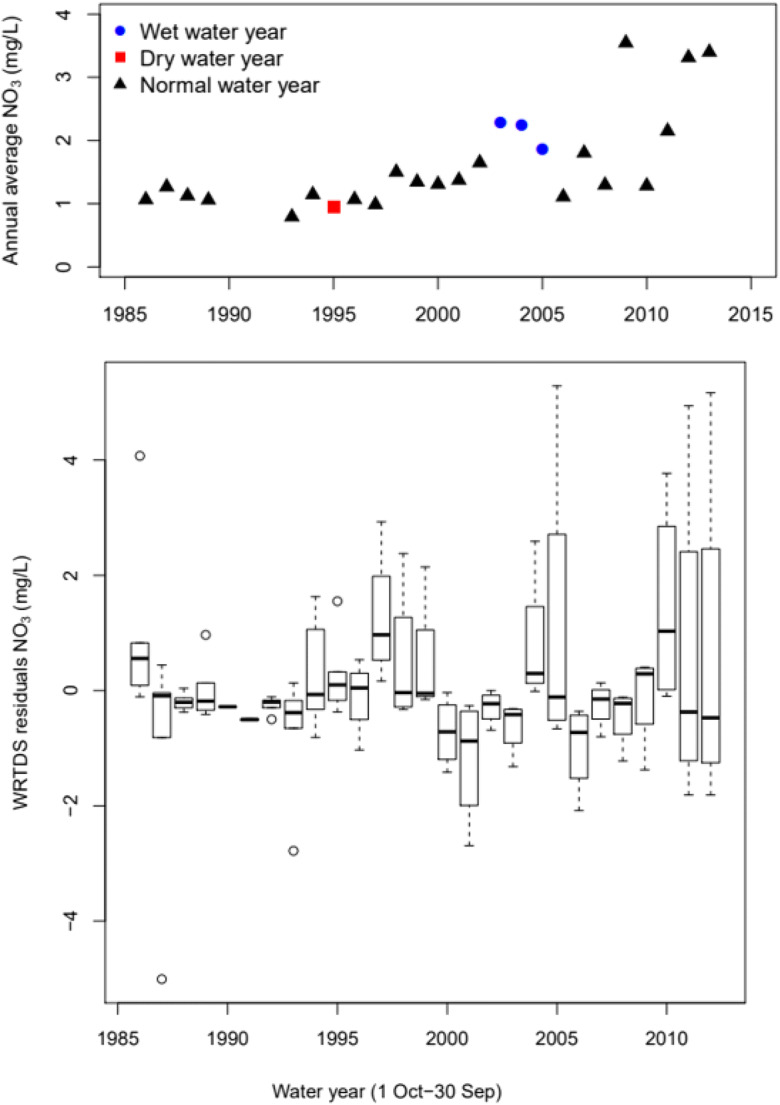
Average annual WRTDS estimated NO_3_ concentrations and WRTDS NO_3_ residual concentrations. Wet and dry years are defined ±1 standard deviation from the mean

**Fig. 14:**
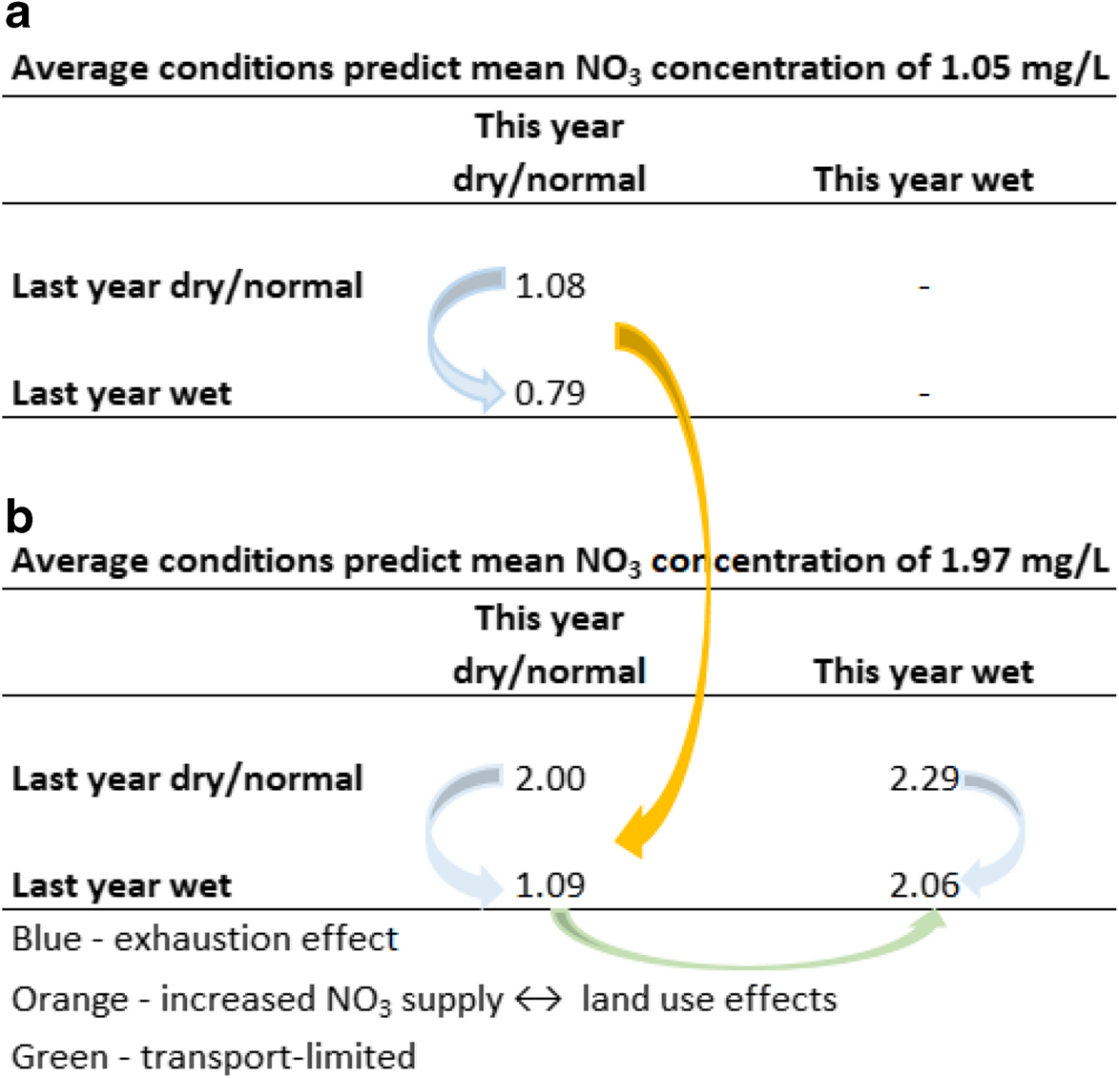
WRTDS average annual NO_3_ concentrations in mg/L. Wet and dry years are defined ±1 standard deviation from the mean

WRTDS C–Q nitrate concentration residuals from 1986 to 2013 are shown in Fig. 13 along with the model estimates of annual average concentrations. An increase in NO_3_ residuals is observed in 1998. From 1998 to 2000, concentration residuals remain positive; however, they alternate from positive to negative thereafter. Years 2001–2004 had negative residuals, 2005–2006 positive, 2007–2010 negative, and 2011–2013 positive residuals. Mean annual concentrations between 1986 and 1997 are lower than that between 1998 and 2013, 1.05 mg/L and 1.97 mg/L, respectively. Previously, we noted that river nitrate concentrations are controlled by antecedent hydrologic conditions (“Effect of antecedent hydrologic conditions on C–Q residuals”). This suggests that there are lag effects in the hydrologic system with prolonged dry conditions limiting NO_3_ transport to the stream while wet events exhausted NO_3_ supplies. Figures 13 and 14 show the lags or ‘memory effect’ in annual average concentrations such that wet conditions of the year before lead to a decrease in concentration in the current year. And dry conditions the year before lead to increased concentration with wet conditions of the current year.

Annual memory effects have been noted before in long-term records (Burt et al. 1988; Burt and Worrall 2009; Worrall et al. 2003; Reynolds et al. 1992). A positive annual memory has been ascribed to a transport-limited situation where there is no shortage of nitrate supply and the impact of a wet year is that nitrate concentrations are greater than expected the following year. Also, the effect of dry conditions is to cause increased nitrate runoff in the year following dry conditions. A negative annual memory effect is associated with supply-limited conditions or an exhaustion effect where wet conditions cause increased nitrate loss so that less nitrate is available for export from the watershed later.

**Fig. 15:**
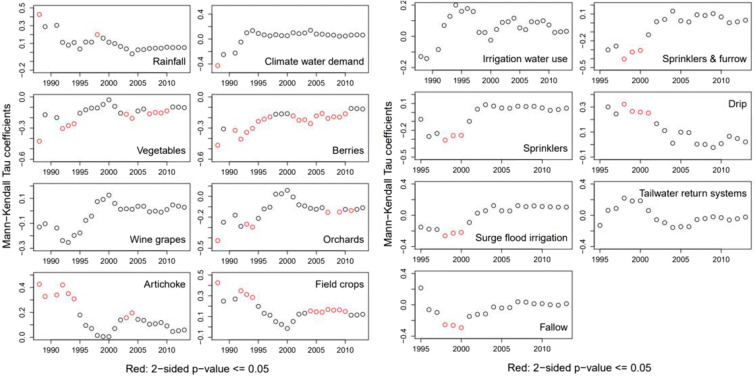
Kendall’s tau coefficients between NO_3_ residuals and rainfall, climate water demand, major crop areas, agricultural water use, irrigation, and conservation areas using expanding window with initial subset set at 3 years, results plotted against the final year of the series

We also examined long-term memory effects in the context of climate and land-use driving variables. Mann-Kendall trend analyses for the NO_3_ C–Q residuals and annual driving variables were performed using an expanding window technique. This was executed such that the initial subset starts at the beginning of the record with an initial length of 3 years; the subset is allowed to expand by 1 year until the end of the time series to identify trends within the time series. Plots of calculated Tau coefficients from the Mann-Kendall analysis of correlations between NO_3_ C–Q residuals, rainfall, climate water demand, major crop areas and irrigation, and conservation practices are shown in Fig. 15.

The expanding window technique is relatively conservative in that events that affect only 1 year of record are severely dampened by that of the other years considered. This means that in an expanding window perspective, only long-term effects will have a significant and lasting effect on the NO_3_ concentration lags. The effects on the concentration lags will diminish if the year-on-year effect was constant. Annual rainfall had significant and positive correlation with NO_3_ C–Q residuals in 1988; thereafter, Tau decreased until increasing sharply in 1997 following dry 1995 conditions and the effect is positive and significant. The effect of dry conditions in 1995 was short-lived, however as shown by the decreasing trend in Tau as well for the rest of the period when the effect of rainfall on NO_3_ C–Q residuals was not significant. Moreover, CWD (rainfall–potential evapotranspiration) had no significant correlation with NO_3_ C–Q residuals for the entire period, though average annual concentrations increased between the period 1986–1997 and 1998–2013 from 1.05 to 1.97 mg/L, respectively. Thus, climate drivers are not solely responsible for this change rather internal watershed dynamics play a critical role.

**Fig. 16:**
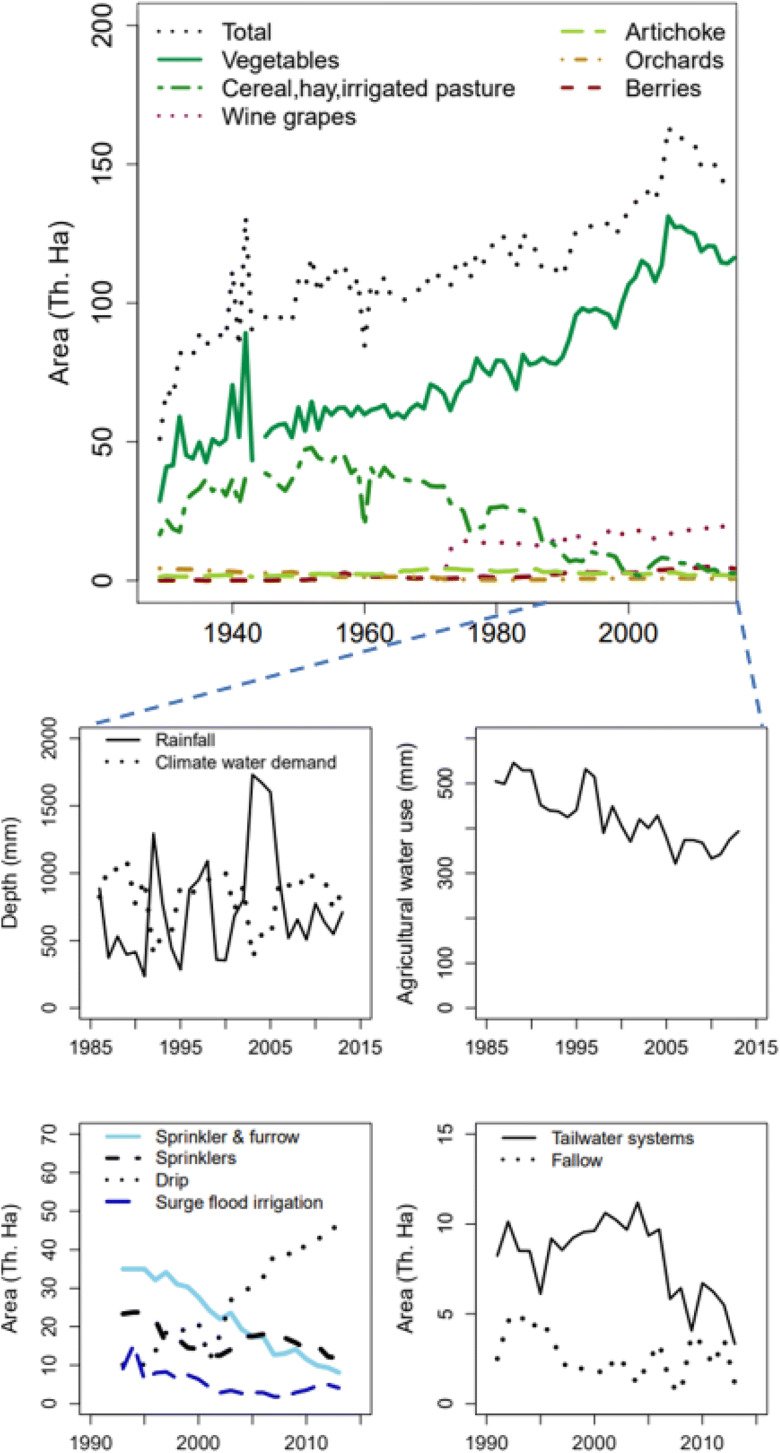
Annual rainfall, climate water demand, land use, and water use. **a** Crop areas. **b** Climate variables. **c** Irrigation water used. **d** Irrigation practices. **c** Conservation practices

Figure 14 indicates possible explanations for lag effects observed in annual average NO_3_ concentrations. Wet conditions have an exhaustion effect on NO_3_ concentrations while dry conditions are transport-limited and the increase in average predicted NO_3_ concentrations between 1986–1997 and 1998–2013 are due to internal factors in the watershed. By the mid-20th century, vegetables became the main crop in the region overtaking field crops—alfalfa, barley, sugar beet, and wheat (Fig. 16). In 1900, field crops were mostly rain-fed and constituted 96% of all those produced; however, by 1950, they declined to only 26% while vegetable crop production accounted for 58% of the region production area. Similarly, wine grape production expanded from 630 to 14,000 ha between 1971 and 1974, mostly on converted field crop and grazing lands.

Results on the lag effects on NO_3_ C–Q residuals of land use and management indicated that changes associated with drip irrigation, annual rainfall, and artichoke and field crop areas had significant and positive effects on NO_3_ C–Q residuals with a memory of 9, 13, and 25 years, respectively. Areas for seasonal vegetables, orchards, and berries had negative and significant on NO_3_ C–Q residuals with lag on these effects of two and a half decades. Irrigation management with a shift towards use of sprinklers, or combination of furrow and sprinkler irrigation had negative and significant effects on NO_3_ residuals with a lag of 10 years while changes in fallow area and areas using surge flood irrigation had negative effects on NO_3_ residuals with memory effect lasting 8 years.

Effects of climate and land use variables at the end of the analysis period (2013) are not significant. However, comparing Tau values, we can extract relative magnitudes and direction of the variable long-term effects on NO_3_ C–Q residuals (Table 8). Mann-Kendall analysis in 2013 yielded Tau values that were nearly zero for most variables except for field crops, surge flood irrigation, seasonal vegetables, berries, and orchards areas. Areas of field crops and surge flood irrigation had positive long-term effects on NO_3_ C–Q residuals—+0.12 and +0.11, respectively; however, these are significant at 94% and 86%, respectively. On the other hand, areas of seasonal vegetables, orchards, and berries had negative long-term effects on NO_3_ C–Q residuals—−0.10, −0.11, and −0.12, respectively; these are only significant at the 89%, 92%, and 93% levels.

**Table 8:** Effects of climate and land use variables at end of the analysis period (2013)

Variable	Lag/memory effect scales (years)	Tau	Two-sided
			*P* value^a^
Field crops (ha)	27	0.12	0.06
Surge flood irrigation (ha)	23	0.11	0.14
Climate water demand (mm)	*27*	*0.06*	*0.31*
Artichoke (ha)	*27*	*0.06*	*0.35*
Rainfall (mm)	*27*	*0.05*	*0.40*
Sprinklers (ha)	*21*	*0.05*	*0.51*
Irrigation water used (mm)	*27*	*0.03*	*0.62*
Sprinklers and furrow (ha)	*23*	*0.03*	*0.68*
Wine grapes (ha)	*27*	*0.03*	*0.64*
Drip (ha)	*21*	*0.02*	*0.71*
Fallow (ha)	*23*	*0.01*	*0.84*
Tail water return systems (ha)	*23*	*−0.02*	*0.75*
Seasonal vegetables (ha)	27	*−*0.10	0.11
Orchards (ha)	27	*−*0.11	0.08
Berries (ha)	27	*−*0.12	0.07

Effects of variable on NO_3_ C–Q residuals nearly zero

Italicized values indicate that effects of variable on NO C-Q residuals 3 nearly zero

***0.001, **0.01, *0.05

^a^Two-sided *p* value ranges: ns > 0.5; 0

^b^Including cereal grain, hay, and irrigated pasture

## Conclusions

Assessment of concentration trends in river salinity at the watershed scale is an initial step and integral part of developing salinity mitigation measures and is critical towards evaluating ecosystem services of the river system. The objective of this study was to assess past behavior of river solute concentrations (regime shifts) and diagnose the underlying controls on river salinity over the 27-year grab sampling record. The power of the analysis carried out lies in piecing together using sparse but long-term grab sampling data the complex factors responsible for solute behavior in the watershed. The results can support water resource planning and water allocation policy evaluation. Historical trends in river salinity offer a basis for understanding changes in state or regime shifts in solute concentration and the time constants of a range of driving processes.

First, we used the rating curve residuals to delineate periods of solute species higher and lower concentration levels (Fig. 7). Residuals are differences between measured concentrations and those expected based on the long-term average expressed by the rating curve can be used to reveal systematic departures in sample C behavior. We then examined the hydrogeochemical processes responsible for the production of different solute concentrations over time in the lower Salinas River. We found that the primary source of major salinity constituents in the lower Salinas River was associated with rock weathering that released Ca, Mg, Na, HCO_3_, and SO_4_ as well ion exchange of Ca, Mg, and Na species. Concentrations of K, NO_3_, and Cl were associated with human activities and agricultural practices were the major source of K and NO_3_ concentrations in the river.

Watershed hydrology had a large control on these concentrations. Over time, the WRTDS concentration–discharge relationships for Ca, Mg, and K indicated constant concentrations over a range of stream discharges. The stability of concentrations emerges because of increased solute mass stores in the watershed; for Ca and Mg, these are associated with rock weathering processes while for K with K-fertilizer inputs. Contributions for all solutes associated with high flows have decreased over time; however, low flow contributions of NO_3_ have increased with time suggesting an emerging problem associated with agricultural practices. There has been increased nitrification activity in the watershed from 1998 to 2013 and changing land use and management drives these changes.

Conceptually, WRTDS C–Q residuals are the portion of the concentration signal not accounted for by contemporaneous discharge, season, or long-term trend. Historical and event-based hydrological characteristics were found to play a significant role in determining solute behavior in the lower Salinas. Baseflow contributions increased concentrations of Ca, Mg, Na, Cl, HCO_3_, and NO_3_ while basin wetness represented by water yield (rainfall-actual evapotranspiration) decreased concentrations for all solute species. Effect of past hydrologic events on current concentrations releases was such that concentrations for all solutes decreased following prolonged dry conditions as well as following periods of mid to high stream flows. Dry conditions over seasonal time scales <100 days decreased HCO_3_ concentrations. Dry conditions over seasonal to annual time scales decreased Ca, K, SO_4_, and NO_3_ concentrations, and dry conditions over seasonal to inter-annual time scales decreased Mg, Na, and Cl concentrations. On the other hand, following wet condition concentrations released to the stream decreased (flushing or washoff). Different ranges of stream flows had the effect of depleting ion stores within the watershed so that concentration exports decreased after wet events. Stream flows between 3 and 90 m^3^/s before sampling decreased concentrations of Ca; stream flows of 3 to 1200 m^3^/s decreased concentrations Mg, Na, and NO_3_; flow between 3 and 1000 m^3^/s decreased SO_4_ concentrations and flows between 2 and 1200 m^3^/s decreased Cl concentrations. Overall, regulated stream flow releases from reservoirs are critical for dilution effects while groundwater contributions to river flows increased solute concentrations.

We focused a section (“Relationship between NO3–Q residuals, climate, and land use variables”) on the dynamics of NO_3_ concentration in the river as this has been identified as a major source of pollution of drinking water in the region. Nitrate concentrations increased with time suggesting an emerging problem associated with agricultural production. River nitrate concentrations were controlled by antecedent hydrologic conditions. Prolonged dry conditions limited NO_3_ transport to the stream while wet events exhausted NO_3_ supplies. A more direct anthropogenic positive trend in NO_3_ that has persisted since the mid-1990s is associated with lag effects of field crops areas and use of flood irrigation (Fig. 15; Table 8). While conversion to drip and sprinkler irrigation has in the long term reduced the effect of farming practices on river NO_3_ concentrations.

This study highlights the importance of understanding shifts in pollution state of a river systems and drivers to which a region may be vulnerable under changing land uses and climate. As human activity continues to modify landscapes, there is a need to understand deeply the consequences of human intervention to better manage for sustainable ecosystem service provisioning. In the Salinas Valley tests on the lag effects associated with the current cropping trends that are dominated by vegetable, berries and wine grapes and use of drip and sprinkler irrigation system will be a required future effort. Additionally, future work should establish thorough monitoring and modeling the linkage between groundwater flow and salinity transport to the stream. Furthermore, there is a need to understand salinity fluxes during storm events. Understanding fluxes during storm events is critical since storms in central California are expected to increase in frequency and intensity as part of ongoing climate change. Transport associated with storm events is difficult to characterize with “grab sampling” approaches because they are infrequent. High-frequency sensors to collect continuous water quality data especially NO_3_ will improve our understanding of the stream water chemistry dynamics during rare and episodic events. This task is especially critical since discharge-solute relationships change during extreme precipitation events or due to variable event-based hysteresis in the solute concentration–streamflow relationship.
